# Modeling the impact of novel diagnostic tests on pediatric and extrapulmonary tuberculosis

**DOI:** 10.1186/1471-2334-14-477

**Published:** 2014-09-03

**Authors:** Claudia M Denkinger, Beate Kampmann, Syed Ahmed, David W Dowdy

**Affiliations:** Beth Israel Deaconess Medical Center, Boston, USA; McGill University, Montreal, Canada; Foundation for Innovative New Diagnostics, Geneva, Switzerland; Imperial College, London, UK; Johns Hopkins Bloomberg School of Public Health, Baltimore, USA

**Keywords:** Tuberculosis, Diagnostics, Pediatrics, Extrapulmonary, Modeling

## Abstract

**Background:**

Extrapulmonary tuberculosis (EPTB) and most pediatric TB cannot be diagnosed using sputum-based assays. The epidemiological impact of different strategies to diagnose EPTB and pediatric TB is unclear.

**Methods:**

We developed a dynamic epidemic model of TB in a hypothetical population with epidemiological characteristics similar to India. We evaluated the impact of four alternative diagnostic test platforms on adult EPTB and pediatric TB mortality over 10 years: (1) Nucleic acid amplification test optimized for diagnosis of EPTB (“NAAT-EPTB”); (2) NAAT optimized for pediatric TB (“NAAT-Peds”); (3) more deployable NAAT for sputum-based diagnosis of adult pulmonary TB (“point-of-care (POC) sputum NAAT”); and (4) more deployable NAAT capable of diagnosing all forms of TB using non-invasive, non-sputum specimens (“POC non-sputum NAAT”).

**Results:**

NAAT-EPTB lowered adult EPTB mortality by a projected 7.6% (95% uncertainty range [UR]: 6.5-8.8%). NAAT-Peds lowered pediatric TB mortality by 6.8% (UR: 4.9-8.4%). POC sputum NAAT, though only able to diagnose pulmonary TB, reduced projected pediatric TB deaths by 13.3% (UR: 4.6-15.7%) and adult EPTB deaths by 8.4% (UR 2.0-9.3%) simply by averting transmission of disease. POC non-sputum NAAT had the greatest effect, lowering pediatric TB mortality by 34.7% (UR: 26.8-38.7), and adult EPTB mortality by 38.5% (UR: 30.7-41.2). The relative impact of a POC sputum NAAT (i.e., enhanced deployability) versus NAAT-EPTB (i.e., enhanced ability to specifically diagnose TB-NSP) on adult EPTB mortality depends most strongly on factors that influence transmission, with settings of higher transmission (e.g., higher per-person transmission rate, lower diagnostic rate) favoring POC sputum NAAT.

**Conclusion:**

Although novel tests for pediatric TB and EPTB are likely to reduce TB mortality, major reductions in pediatric and EPTB incidence and mortality also require better diagnostic tests for adult pulmonary TB that reach a larger population.

**Electronic supplementary material:**

The online version of this article (doi:10.1186/1471-2334-14-477) contains supplementary material, which is available to authorized users.

## Background

Improved diagnostic tests are needed to reduce the tremendous burden of morbidity and mortality due to tuberculosis (TB), a disease that affects 8.6 million people and kills 1.3 million every year [1]. In 2010, Xpert MTB/RIF (“Xpert”), a high-sensitivity rapid molecular test, was released and soon thereafter endorsed by the World Health Organization (WHO) for the diagnosis of adult pulmonary TB [2–4].

While pulmonary TB accounts for the largest burden of disease and Xpert as well as most other TB diagnostics are designed to use sputum as a biological specimen, at least 20% of all adults (up to 30-40% in HIV patients) – and most children with TB – either cannot produce sufficient sputum (“sputum-scarce”), do not have sufficient bacilli in their sputum to be detected, or have extrapulmonary TB (EPTB) [5–8]. EPTB and pediatric TB result in significant morbidity and mortality dependent on the organs affected (e.g., central nervous system) and due to the difficulty in achieving a diagnosis [9, 10]. Therefore, further research on improving existing tests and developing novel tests for pediatric TB and EPTB is necessary.

The most recent WHO guideline recommends Xpert for use in children and individuals with certain subsets of EPTB [11, 12]. However, the evidence base for this recommendation is considered to be very low-quality, and the accuracy of Xpert in its current version is insufficient for subsets of EPTB (e.g. pleural TB and TB meningitis) [11, 12].

In developing novel tests for pediatric and EPTB, one approach could be an “optimized” Xpert or other nucleic acid amplification test (NAAT) capable of detecting *M. tuberculosis* with higher sensitivity in specimens other than sputum (e.g. tissue) for example through improved sample processing and DNA extraction. Another approach targeting the diagnosis of pulmonary TB in children and others who cannot produce good sputum, might be an improved non-sputum based assay (possibly optimized Xpert or other NAAT) using more easily accessible specimens (e.g. nasopharyngeal samples) [11, 13].

A third approach to improving the control of pediatric and EPTB is to develop more deployable tests for adult pulmonary TB, reasoning that children and immunocompromised individuals are at highest risk of developing active TB from recent transmission, and diagnosis and treatment strategies capable of reducing TB transmission in a community might have important indirect effects on pediatric and extrapulmonary TB [14]. The ideal assay, however, would be a test with improved diagnosis for pulmonary TB, “sputum scarce” TB, and EPTB in adults and children alike using clinical specimens other than sputum (e.g., blood or urine) on a deployable (able to be rolled out at microscopy center level) and sensitive platform linked to rapid treatment initiation [15].

To evaluate the comparative effectiveness of such different diagnostic approaches for EPTB and pediatric TB and evaluate them against a current baseline scenario with smear microscopy and alternatively with Xpert for adult pulmonary TB at the district level health care, we constructed a dynamic epidemic model of TB in a generalizable population, estimating ten-year EPTB and childhood TB incidence and mortality if four hypothetical but emblematic tests for EPTB and pediatric TB could be implemented.

## Methods

### Model structure

**Figure 1:**
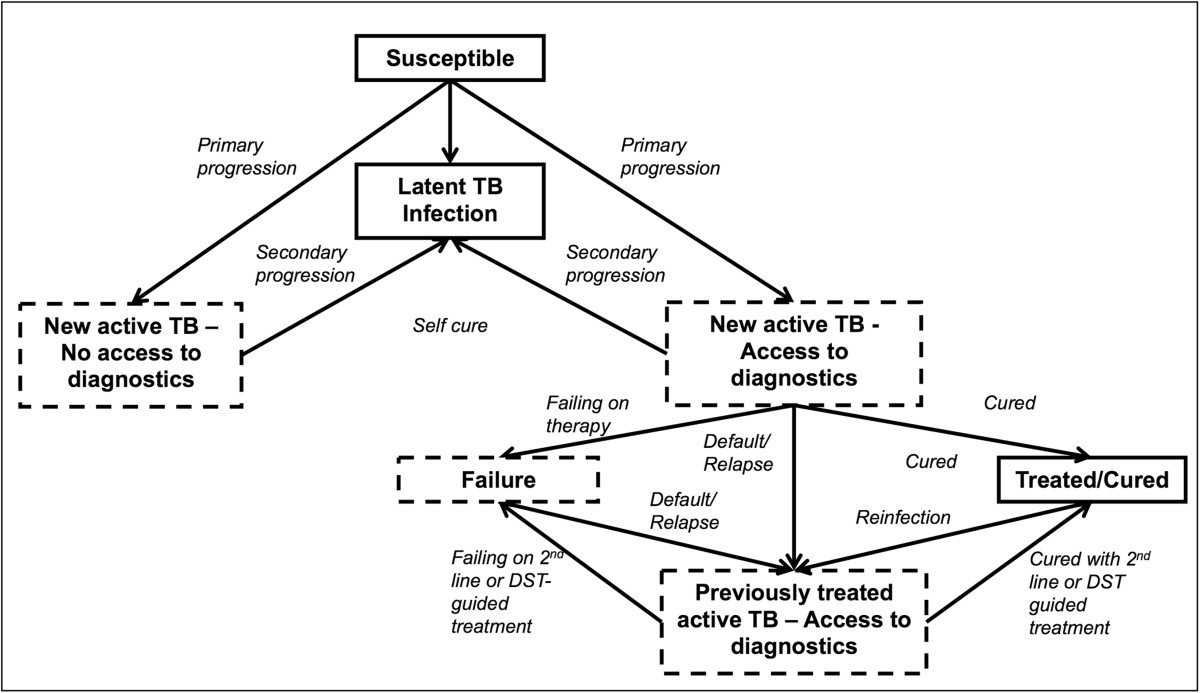
**Study flow diagram.** Dashed boxes contain subjects that are infectious. The compartments are also defined by the individual’s age, HIV status, the type of tuberculosis (TB – pulmonary or extrapulmonary) and by the TB drug susceptibility pattern (sensitive, isoniazid [INH]-monoresistant, multidrug-resistant [MDR], and extensively-drug resistant [XDR]); these delineations are not shown in the diagram for simplicity.

We built a compartmental model using ordinary differential equations to describe a mature tuberculosis epidemic in a stable, homogeneously mixing population structured by age and HIV status. Figure 1 describes the basic structure of the model; a more detailed description is found in the supplement. Given that the hypothetical assays we evaluate should have impact across the epidemiological contexts of greatest TB burden, we modeled a paradigmatic population with an incidence of drug-susceptible (181/100,000 per year) and drug-resistant TB (2.1% MDR among new cases) and an HIV prevalence similar to that of India (0.3%) [16]. We used data from World Health Organization (WHO) notifications and other published literature to inform parameters in our model (Table 1) [17, 18]. In order to optimize the benefit of tests for pediatric TB relative to those for adult pulmonary TB, we made liberal assumptions about the percentage of TB that occurs in children (taking into account the likely substantial underreporting of pediatric TB) [19–29].

**Table 1:** **Parameters**

Definition	HIV status	Value	Range	References
Non-TB death rate per year	all	0.022	0.02-0.025	
TB mortality per year	HIV negative	0.15	0.1-0.22	[1, 19]
	HIV positive	0.50	0.4-0.7	[20]
Transmission events per infectious person-year in year 10*	all	8.95		
Partial immunity afforded by previous infection	HIV negative	0.45	0.4-0.55	[21, 22]
	HIV positive	0	0-0.2	
Proportion of TB infections progressing rapidly to active TB	HIV negative	0.14	0.05-0.14	[23]
	HIV positive	0.25	0.16-0.27	
Endogenous reactivation rate per year	HIV negative	0.0005	0.08-1.4 x10^−3^	[24]
	HIV positive	0.05	0.03–0.05	
Rate of self-cure in active TB per year	HIV negative	0.1	0.08-0.28	[25]
	HIV positive	0	0-0.2	
Percent of patients without access to diagnostics	all	0.1	0.05-0.25	[26]
Sensitivity of current diagnostic standard for PTB	all	0.80	0.6-0.9	[18, 33]
Sensitivity of current diagnostic standard for EPTB	all	0.6	0.4-0.8	
Sensitivity of novel test methods for PTB	all	0.95	0.75-0.98	[27–29]
Proportion of adults that develop EPTB or sputum scarce PTB in	HIV negative	0.18	0.15-0.25	[31, 32]
	HIV positive	0.35	0.3-0.7	
Proportion of children that develop EPTB or sputum scarce PTB independent of HIV status (weighted average among different age groups)	all	0.85	0.6-0.9	[5]

*Abbreviations*: *TB* tuberculosis, *NAAT* nucleic-acid amplification test, *EPTB* extrapulmonary *TB*. *averaged over all patients with pulmonary TB (HIV-positive patients are estimated to be about half as infectious as *HIV*-negative patients).

Our primary modeling aim was to assess the maximum potential impact of a test that has enhanced capacity to diagnose clinical manifestations of TB that are not readily diagnosable with sputum-based tests. Our primary outcomes were the projected incidence and mortality due to TB overall, pediatric TB, and adult EPTB.

Pediatric and extrapulmonary TB are heterogeneous entities, including clinical sites as diverse as lymph nodes, bone, and the central nervous system, each with different degrees of severity and ability to be diagnosed by currently available means. As no model can fully account for such clinical diversity, we represent these heterogeneous manifestations as a single entity, labeled “TB with no sputum production” (TB-NSP), that reflects a weighted average of all TB clinical manifestations that are not readily diagnosable by sputum-based assays. We presume that the proportion of active TB consisting of TB-NSP is 85% in children (age 0–15) [6, 30], of which about 70% is in fact pulmonary TB (with and without extrapulmonary components) that cannot be diagnosed because a diagnostic sputum sample cannot be obtained, and the remaining 30% is EPTB. In the adult population, we assume TB-NSP constitutes about 18% in HIV-uninfected adults and 35% in HIV-infected adults (Table 1) [6, 31, 32].

### Model calibration

We first established a baseline “year zero”, modeled as a scenario representative of the current TB epidemic in India [18]. We initiated the model at steady state 65 years prior to year zero (e.g., 1950, if year zero corresponds to 2015), calibrating the TB transmission rate (number of secondary infections per smear-positive person-year) to match India’s WHO-estimated TB incidence (181 per 100,000/year in 2011) [18]. To provide a realistic epidemic trajectory, we reduced the overall TB transmission rate to a degree sufficient to generate a 2% per year decline in TB incidence, the globally estimated average, five years before introducing the diagnostic interventions [18].

### Diagnostic algorithms

At baseline, we assumed a “standard” diagnostic approach for individuals suspected of having adult pulmonary TB; this approach may consist of sputum examinations, ancillary diagnostic tests (e.g. chest X-ray, antibiotic trials), and clinical judgment [33]. We calibrated the sensitivity of this “standard approach” to a value (i.e. 80%) that provided a reasonable estimate of TB case detection rate (model value 70 = Indian national estimate for smear-positive cases) [17]. Diagnosis through this standard approach is assumed to occur at a given constant rate; in calculating this rate, we assumed that the delay in diagnosing TB-NSP would be twice that for adult pulmonary TB because of the difficulty in obtaining a sample from the site of infection (e.g. pleural aspirate or gastric fluid) for diagnosis [34].

In our analytic scenarios, we then enhanced this baseline diagnostic algorithm with platforms designed to improve diagnosis of TB-NSP through different combinations of (a) optimized detection of TB using clinical specimens other than sputum and/or (b) enhanced deployability of the assay itself:

### Xpert MTB/RIF for adult pulmonary TB

Starting in year zero, we augmented the “standard” diagnostic approach with Xpert for adult pulmonary TB for adult pulmonary TB. We assumed that Xpert would increase the overall sensitivity of the standard approach for the diagnosis of adult pulmonary TB – incorporating all existing diagnostic tests, plus clinical judgment – from 80% to 95% (i.e., detecting 75% of TB cases who would otherwise be missed with 98% specificity) [33, 35]. We assumed that given Xpert’s current infrastructure requirements (e.g., constant power supply), it would be implemented in district level health centers and therefore would reach 15%, 30% and 30% of new, previously treated, and failure cases respectively (i.e. deployability limited to district level health centers). The remainder of cases continued to be diagnosed with the standard approach.

### Optimized diagnostic approaches

We compared the impact of Xpert for adult pulmonary TB to that of four hypothetical tests that were designed to illustrate important test characteristics (e.g., components of a “target product profile”) and tradeoffs between deployability and ability to diagnose TB-NSP (and may or may not involve an Xpert like algorithm). We use the term “deployability” to denote the level of the healthcare system at which a diagnostic test can be implemented, and thus the proportion of the healthcare-seeking population that could reasonably be tested – assuming that tests implemented at more peripheral levels (e.g., microscopy centers) could reach a wider population than those implemented only in centralized laboratories. To maintain generalizability, we did not attempt to model any specific test currently in development. The four hypothetical tests were:

1. “NAAT Peds” – same accuracy (i.e. 95% sensitivity and 98% specificity), deployability and ability to diagnose adult pulmonary TB as Xpert but capable of diagnosing, in addition, 70% of all forms of TB-NSP with a respiratory component in children (e.g., using nasopharyngeal fluid but not able to diagnose, for example, TB meningitis);
2. “NAAT-EPTB” – deployability equal to Xpert (owing to the need for obtaining invasive specimens such as cerebrospinal fluid or gastric fluid) but capable of diagnosing both TB-NSP and pulmonary TB with the same accuracy as Xpert (i.e. 95% sensitivity and 98% specificity), through use of non-respiratory samples;
3. “POC sputum NAAT” – same sensitivity and specificity as Xpert for adult pulmonary TB, only more portable and less dependent on existing infrastructure (i.e. deployable at microscopy center level); modeled as reaching 50%, 80%, and 100% of new, previously treated, and failure cases respectively;
4. “POC non-sputum NAAT” – similar to POC sputum NAAT (same sensitivity and specificity as Xpert) but using a more accessible clinical specimen (e.g., urine or finger-prick blood) and thus capable of diagnosing both pulmonary TB and TB-NSP, with the same speed for both as it does not require an invasive sample but without rifampin resistance detection [14, 15].

### Sensitivity analysis

We conducted one-way sensitivity analyses on all model parameters taking as the outcome the difference in adult EPTB mortality comparing NAAT-EPTB (improved detection of TB-NSP through access of non-pulmonary sites) to POC sputum NAAT (improved detection through higher deployability of a sputum-based test). The ranges of the parameters are based on the available literature and possible advances in the near future, as outlined in Additional file 1: Table S1. To estimate variability associated with simultaneous changes in all parameters, we also conducted a probabilistic uncertainty analysis using Latin Hypercube Sampling (Additional files 1 and 2 detail in the supplement).

## Results

### Impact on TB incidence

In the absence of any improvement in TB diagnosis, we projected, in year ten, 44.8 TB cases and 9.4 deaths per 100,000 in children and 89.5 cases and 22.8 deaths in adults (Table 2). If Xpert for adult pulmonary TB was scaled up in year zero for 15% of new and 30% of previously treated cases, TB incidence fell from this baseline by 5.0% in children (95% uncertainty range [UR]: 1.6-6.1%) and 4.0% in adults (UR 1.0-4.5%). Implementation of Xpert for adult pulmonary TB after 10 years did not change the proportion of TB that was TB-NSP (30% in adults, 78% in children).

**Table 2:** **Projected tuberculosis outcomes**

	10-year projected tuberculosis outcomes								
	Incidence per 100,000			Prevalence per 100,000			Mortality per 100,000		
	Adult total	Adult EPTB	Children	Adult total	Adult EPTB	Children	Adult total	Adult EPTB	Children
	N (% reduction*)	N (% reduction*)	N (% reduction*)	N (% reduction*)	N (% reduction)	N (% reduction*)	N (% reduction*)	N (% reduction*)	N (% reduction*)
**Existing standard**	89.5 (Ref)	13.9 (Ref)	44.8 (Ref)	114.8 (Ref)	34.4 (Ref)	62.8 (Ref)	22.8 (Ref)	7.3 (Ref)	9.4 (Ref)
**Xpert for adult pulmonary TB**	86.0 (4.0%)	13.4 (3.7%)	42.6 (5.0%)	109.3 (4.8%)	33.2 (3.5%)	59.8 (4.9%)	21.8 (4.5%)	7.1 (3.0%)	8.9 (4.8%)
**NAAT-** **Peds**	86.0 (4.0%)	13.4 (3.7%)	42.6 (5.0%)	109.2 (4.9%)	33.1 (3.9%)	58.5 (6.9%)	21.8 (4.5%)	7.1 (3.3%)	8.7 (6.8%)
**NAAT-** **EPTB**	86.0 (4.0%)	13.4 (3.7%)	42.6 (5.0%)	107.8 (6.1%)	31.7 (7.9%)	58.0 (7.7%)	21.4 (5.9%)	6.7 (7.6%)	8.7 (7.6%)
**POC NAAT sputum**	79.6 (11.1%)	12.5 (10.2%)	38.6 (13.8%)	99.8 (13.0%)	31.0 (9.8%)	54.4 (13.4%)	20.0 (12.2%)	6.7 (8.4%)	8.1 (13.3%)
**POC NAAT non-** **sputum**	80.2 (10.4%)	12.6 (9.6%)	39.0 (13.0%)	90.5 (21.2%)	21.1 (38.8%)	41.0 (34.7%)	18.0 (21.3%)	4.5 (38.5%)	6.1 (34.7%)

*Abbreviations*: *POC* point of care, *NAAT* nucleic-acid amplification test, *EPTB* extrapulmonary *TB*. *compared to existing standard = Ref. Incidence, prevalence and mortality for children and adults by year ten with different interventions.

**Figure 2:**
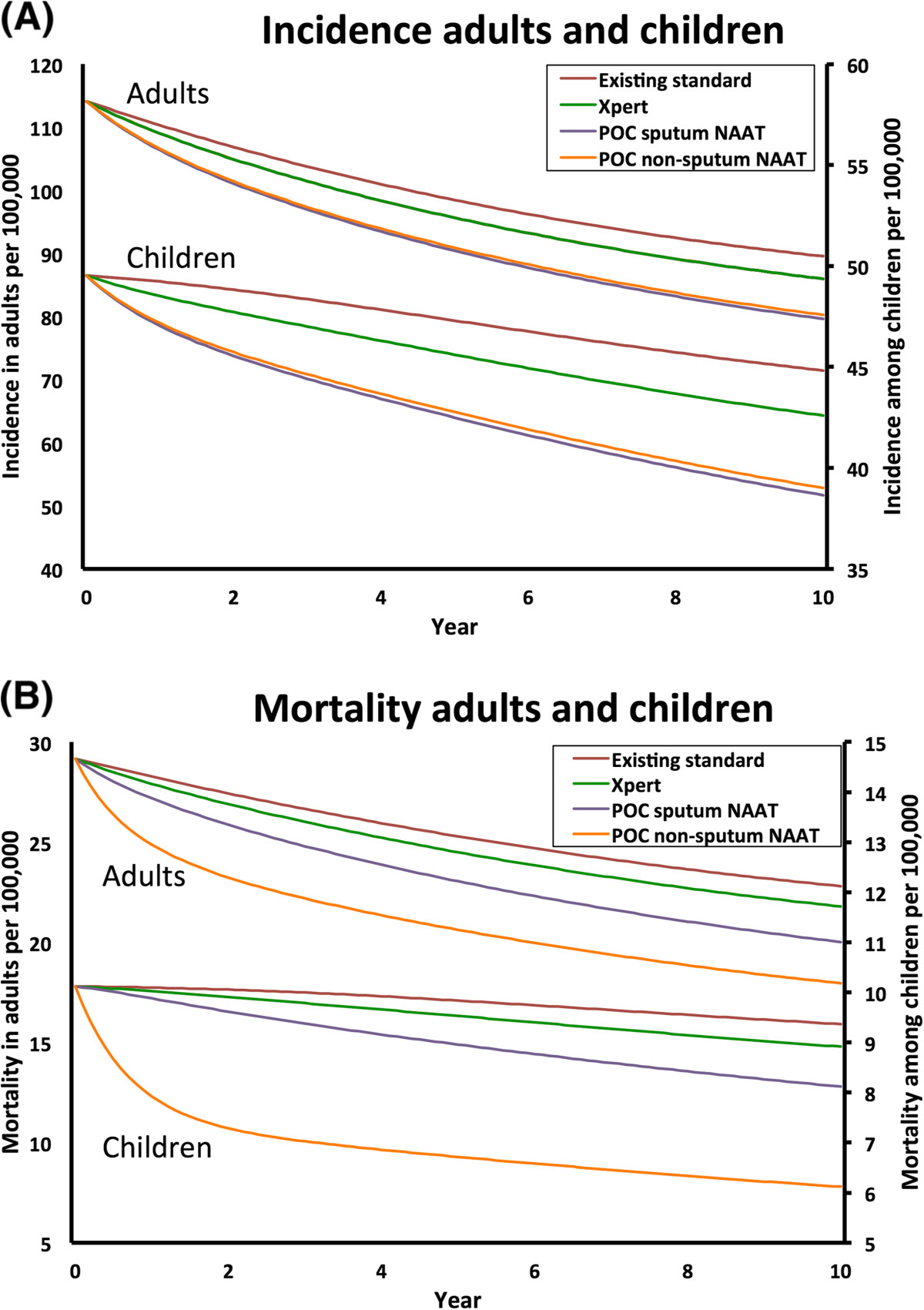
**Impact of different tests on pulmonary and extrapulmonary TB (A) incidence and (B) mortality in adults and children.** Trajectory of **(A)** overall tuberculosis (TB) incidence and **(B)** mortality over 10 years without further intervention (maroon line), with introduction of Xpert for adult pulmonary TB (green line; coverage 15%, 30%, 30% among new, previously treated and failure cases) and introduction of POC sputum NAAT (purple line; coverage of 50%, 80%, 100%). In addition, we project the incremental impact of POC-non-sputum NAAT that was optimized for detection of both pulmonary TB and extrapulmonary TB (EPTB) (orange line) using non-invasive samples thus eliminating a delay in diagnosis of EPTB and being deployed at the same level of coverage as POC sputum NAAT.

Replacement of Xpert for adult pulmonary TB with POC sputum NAAT (i.e., a more deployable but equally accurate sputum-based test) resulted in more TB being diagnosed and treated, thus reducing pediatric TB incidence by 13.8% (UR 4.6-16.3%) and adult TB incidence by 11.1% (UR 2.7-11.9%). A similar test on non-sputum-based samples that could not detect rifampin resistance (i.e., POC non-sputum NAAT) had nearly similar impact on incidence: 13.0% (UR 4.0-15.5%, from 44.8 to 39.0 cases per 100,000/year) reduction in children and 10.4% (UR 2.3-11.4%; from 89.5 to 80.2 cases per 100,000/year) in adults (Figure 2A, Table 2). In contrast, NAAT-EPTB and NAAT-Peds did not reduce TB incidence beyond the effect of Xpert for adult pulmonary TB alone (Table 2), reflecting model assumptions that EPTB is not infectious and TB in children is substantially less infectious than pulmonary TB in adults. NAAT-EPTB also had only a small incremental effect on adult TB-NSP prevalence over Xpert alone (4.5% reduction).

### Impact on TB mortality

**Figure 3:**
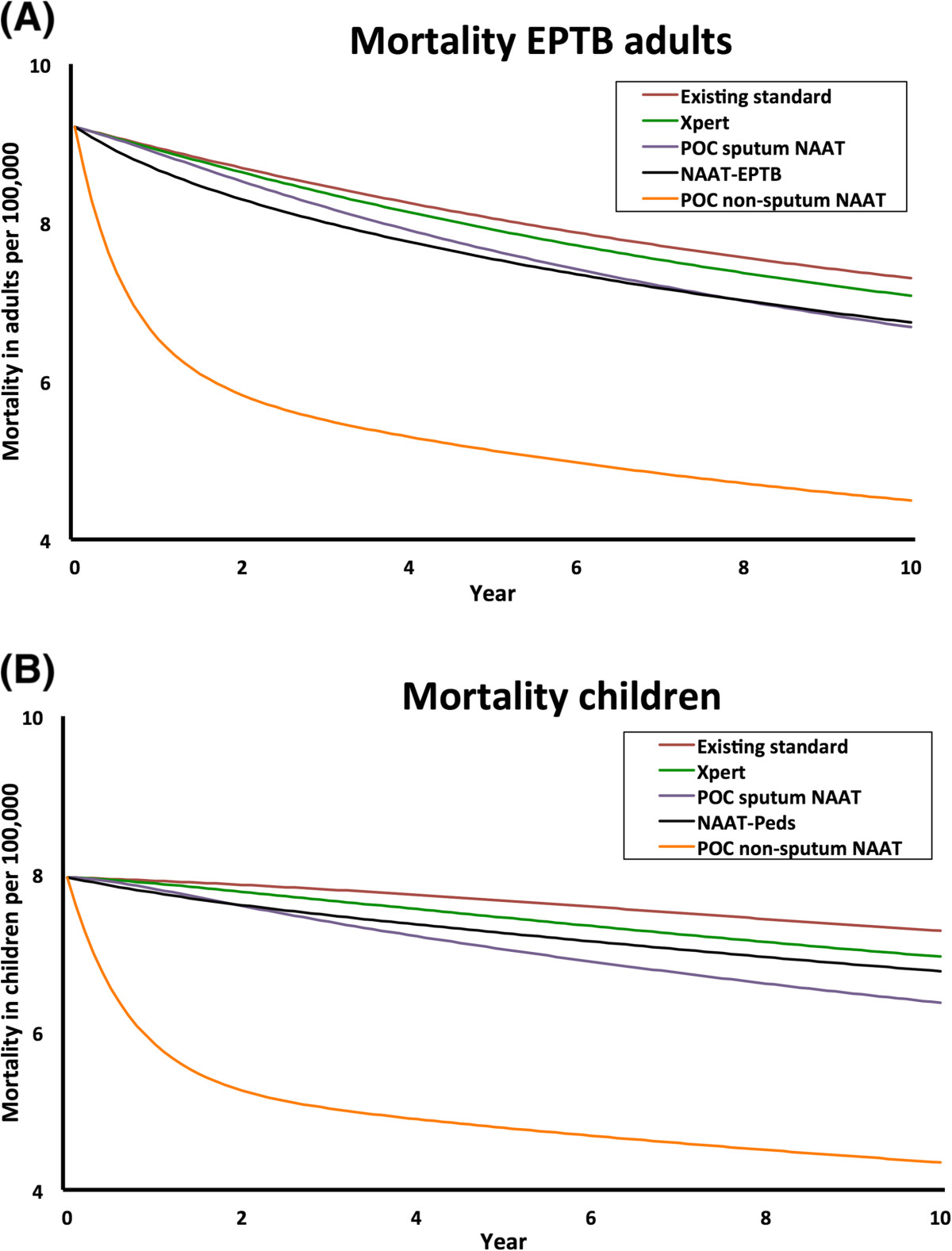
**Impact of different tests on mortality in (A) adult extrapulmonary and (B) pediatric tuberculosis.** Trajectory of extrapulmonary tuberculosis (TB) mortality in adults **(A)** and overall TB mortality in children **(B)** over 10 years without further intervention (maroon line), with introduction of Xpert for adult pulmonary TB (green line; coverage 15%, 30%, 30% among new, previously treated and failure cases), POC sputum NAAT (purple line; coverage of 50%, 80%, 100%), NAAT-EPTB (black line in **A**) and NAAT-Peds (black line in **B**; the latter two both at the same coverage as Xpert). In addition, we project the incremental impact of POC-non-sputum NAAT (orange line).

After 10 years, Xpert for adult pulmonary TB reduced deaths by a projected 4.8% in children (UR 1.6-5.9%) and 4.5% in adults (UR 1.6-5.6%) (Figure 2B, Table 2), closely mirroring reductions in incidence. All diagnostic tests for TB-NSP had substantially greater impact on TB mortality than on incidence. The addition of detection of TB-NSP in children with NAAT-Peds and in adults with NAAT-EPTB enhanced mortality reductions to 6.8% (UR 4.9-8.4%) in children and 5.9% (UR 3.9-7.3%) in adults, respectively.NAAT-EPTB and POC sputum NAAT achieved similar reductions in adult EPTB mortality after 10 years (7.6% [UR 6.5-8.8%] and 8.4% [UR 2.0-9.3%], respectively), but through different mechanisms: NAAT-EPTB resulted directly in better treatment of adult EPTB, whereas POC sputum NAAT reduced TB transmission and thus the number of future cases of adult EPTB. Since its mechanism was more direct, NAAT-EPTB achieved its effect on mortality more rapidly (Figure 3A). However, since the majority of TB is adult pulmonary TB, POC sputum NAAT had a much greater effect on overall adult TB mortality (12.2% reduction [UR 4.5-14.8%] compared to 5.9% [UR 3.9-7.3%] with NAAT-EPTB). Similar to the NAAT-EPTB for adult EPTB mortality, the NAAT-Peds outperformed POC sputum NAAT in early years, but POC sputum NAAT led to greater reductions in TB mortality – and even pediatric-specific TB mortality – over time: 13.3% [UR 4.6-15.7%] versus 6.8% [UR 4.9-8.4%] at ten years (Figure 3B).

POC non-sputum NAAT had the greatest impact on TB mortality, reducing pediatric TB mortality by 34.7% (UR 26.8-38.7%; from 9.4 to 6.1 deaths per 100,000/year) and adult EPTB mortality by 38.5% (UR 30.7-41.2; from 7.3 to 4.5 deaths per 100,000/year).

### Sensitivity analysis

**Figure 4:**
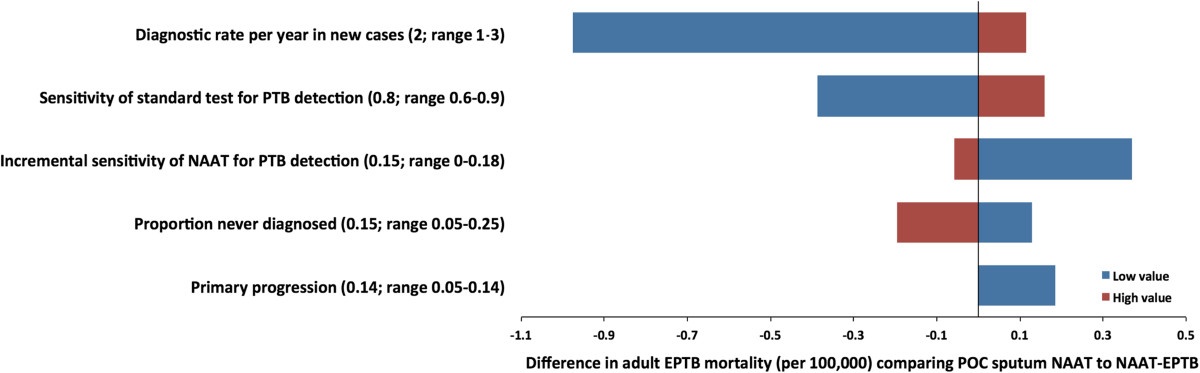
**Sensitivity analysis.** Absolute difference in extrapulmonary tuberculosis (EPTB) mortality in adults per 100,000 by year 10 if POC sputum NAAT is compared to NAAT-EPTB varying one parameter at the time. Numbers in parentheses indicate parameter values at base case and the range from lower and upper end over which the respective parameter is varied (while other parameters are kept constant). The analysis shows that effect of POC sputum NAAT is primarily dependent on reducing transmission of adult pulmonary TB (PTB) and the sensitivity of the test for existing standard for PTB in conjunction with the rate at which the test is used.

We focused our primary sensitivity analysis around the comparison of POC sputum NAAT (i.e., enhanced deployability) versus NAAT-EPTB (i.e., enhanced ability to specifically diagnose TB-NSP). The relative impact of these two tests on adult EPTB mortality depends most strongly on factors that influence transmission, with settings of higher transmission (e.g., higher per-person transmission rate, lower diagnostic rate) favoring POC sputum NAAT (Figure 4). Since the sensitivity of the existing diagnostic standard is much higher for adult pulmonary TB than for TB-NSP, the impact of POC sputum NAAT depends more strongly on the sensitivity of the existing standard than does NAAT-EPTB. No one-way parameter variation across the ranges in Additional file 1: Table S1 changed the absolute difference in mortality by more than 1.2 cases per 100,000 per year. Further sensitivity analyses, including a 3-way sensitivity analysis on the sensitivity of the existing standard for PTB, the incremental sensitivity of the novel test and the diagnostic rate, are reported in the supplement.

## Discussion

This transmission model of a TB epidemic in a defined population suggests that novel assays capable of diagnosing TB-NSP in addition to TB that is diagnosed through sputum examination may generate important (3-8%) reductions in adult EPTB and childhood TB mortality by ten years. However, greater impact on pediatric TB and EPTB mortality (10-15%) may be achievable by deploying tests capable of detecting adult pulmonary TB more widely (i.e., by reducing pediatric and extrapulmonary TB indirectly through reducing transmission). Nevertheless, dramatic reductions in incidence and mortality are unlikely unless a novel test can be developed that cannot only detect TB-NSP but do so using a deployable platform on clinical specimens other than sputum (e.g., hypothetical urine or finger-prick blood assay).

The indirect effect of a more deployable Xpert-like sputum test on pediatric TB (i.e. POC sputum NAAT) on children is particularly noteworthy. TB in children is acquired predominantly from adults and very young children have a greater risk for progression to active disease [36]. Thus, infection and disease in young children are a measure of TB transmission, and tests (e.g., POC sputum NAAT) that add no direct benefit to the diagnosis of TB in these children may counter intuitively still have their greatest effect among such young children, in whom nearly all active TB results from recent transmission [37]. Comprehensive control strategies for pediatric TB should therefore consider that control of pediatric TB requires better tools for diagnosis of the adult pulmonary manifestations responsible for most transmission. Nevertheless, reductions in pediatric TB incidence through reduced transmission are unlikely to be immediate; as such, a specific test for pediatric TB – which can save lives more immediately, and more directly – remains a high priority. An optimized diagnostic and preventive strategy for pediatric TB would include a more sensitive test for pediatric forms of TB plus a more deployable test for adult pulmonary TB that could both reduce the infectious duration and hasten contact investigations in which pediatric contacts of adult TB cases could be given preventive therapy. Our model uses hypothetical tests, however, efforts are ongoing to optimize Xpert for extrapulmonary specimens, develop an automated NAAT that can be deployed at the microscopy center level and identify biomarkers in urine, blood, breath or other more easily accessible samples to make a POC non-sputum test a reality [12, 38, 39].

Prior models of Xpert for adult pulmonary TB have projected a larger impact, specifically on mortality [40]. Our model differs from those models in that we conceptualize a “diagnostic attempt” not simply as the combined sensitivity of a series of tests, but rather as a clinical decision-making process that incorporates ancillary data (e.g., change in symptoms over time) and therefore often occurs on a slower time scale, but with increased overall sensitivity. This higher sensitivity – including clinical or empiric diagnosis – appears to reflect diagnostic reality, at least in settings with trained clinicians and some ancillary testing (e.g., chest X-ray) available [33]. As we incorporate clinical/empiric diagnosis of TB, adding a single diagnostic test to the overall diagnostic pathway results in a lower incremental benefit (and as shown in the supplement a lower cost-effectiveness).

Our model, as with any mathematical representation, has certain limitations. In order to increase transparency and generalizability, the model uses a hypothetical population and is only calibrated to key input parameters reflective of the current TB epidemiology in a population representative of India. This model, therefore, does not account for the complexity of the epidemiological scenario in India or any other single specific location [17, 18, 41, 42]. The model structure also cannot fully capture the heterogeneity of TB epidemics (for example, those driven primarily by HIV) and the complexity of a diagnostic ecosystem with a large, poorly functioning private sector alongside the public sector as present in India [42–44]. Furthermore, the amount of overtreatment in children is also considered to be sizeable but poorly defined. A more accurate test could curb overtreatment and result in more appropriate diagnosis and treatment, potentially improving effectiveness beyond that estimated here. By excluding the potential benefit of limiting overtreatment, we may underestimate the effectiveness of testing in children in these settings.

## Conclusions

In conclusion, diagnostic tests for pediatric TB and EPTB remain a key research priority, as they are likely to have substantial additive impact on mortality over current diagnostic tests that perform insufficiently. These tests are expected to have large market potential. Nevertheless, in the long run, the most effective way to reduce mortality from TB-NSP (and especially pediatric TB, which is highly correlated with recent TB transmission) may be to deploy diagnostic tests and other strategies capable of reducing TB incidence as a whole. One such mechanism is to prioritize tests that can be run on accessible clinical specimens (e.g., blood, urine) and systems to link individuals who test positive directly to treatment. New diagnostic tests are an essential component in reducing the tremendous burden of pediatric and extrapulmonary TB, but elimination of this burden will require a combined approach that also emphasizes reduction in TB transmission and rapid linkage to care.

## Electronic supplementary material

Additional file 1: Table S1: Supplementary information on model structure, parameters and additional analyses. (DOCX 356 KB)

Additional file 2: Figure S1: Sensitivity analysis: comparing the Existing Standard (ES) with NAAT EPTB. (TIFF 315 KB)

Below are the links to the authors’ original submitted files for images.

Authors’ original file for figure 1

Authors’ original file for figure 2

Authors’ original file for figure 3

Authors’ original file for figure 4

Authors’ original file for figure 5

Authors’ original file for figure 6
