# Supplementary material for: Modeling the impact of novel diagnostic tests on pediatric and extrapulmonary tuberculosis
Source: BMC Infect Dis. 2014 Sep 3;14:477. doi: 10.1186/1471-2334-14-477 (PMC4168123; doi:10.1186/1471-2334-14-477)
Supplement: Supplementary file 1 — Additional file 1: Table S1: Supplementary information on model structure, parameters and additional analyses. (DOCX 356 KB) [file 12879_2014_3797_MOESM1_ESM.docx]

**Impact of novel diagnostic tests for childhood tuberculosis and extrapulmonary tuberculosis**

**- Supplementary information**

Claudia M. Denkinger, Beate Kampmann, Syed Ahmed, David W. Dowdy

1. **Model Structure Description**

We constructed a compartmental differential-equation model to describe a mature tuberculosis (TB) epidemic in a stable population of 100,000 children and adults patterned on that of India. The model population was divided into compartments defined by the individual’s age, TB status, type of TB disease, HIV status and TB drug susceptibility pattern (sensitive, isoniazid [INH]-monoresistant, multidrug-resistant [MDR], and extensively-drug resistant [XDR]) (Table E3).

All individuals at any stage of TB infection are presumed to harbor a ‘dominant’ TB strain; this strain determines the patient’s drug-susceptibility pattern upon development of active TB. An individual’s risk of becoming infected with a specified TB strain (defined by drug resistance: sensitive, INH-monoresistant, MDR, or XDR) is directly proportional to the number of active TB patients harboring the specified strain at a given time, and the relative infectivity of that strain. Upon infection, the infecting strain will become the dominant strain in 100% of previously uninfected individuals, and a smaller proportion of individuals harboring latent TB infection because latent infection provides partial protection against reinfection [[1](#_ENREF_1), [2](#_ENREF_2)] (Table E1).

Among individuals in whom the infecting strain becomes the dominant strain, a proportion will progress rapidly to active TB, and the remainder will become latently infected with the new strain [[2](#_ENREF_2)]. Latently infected individuals remain at risk of endogenous reactivation with the same or reinfection with any other strain throughout their lifetimes (taking into account partial protection through prior infection) [[1](#_ENREF_1), [3](#_ENREF_3), [4](#_ENREF_4)]. The risk for primary progression and reactivation depends on the HIV status of the patient (Table E1). Treatment for latent TB infection is not incorporated into the model.

Individuals with HIV co-infection are presumed to have higher baseline mortality than non-HIV infected patients and a higher mortality when infected with TB (Table E1) [[5](#_ENREF_5), [6](#_ENREF_6)]. Furthermore, these patients are presumed not to have a protective effect from latent infection and are more likely to reactivate latent infection [[1](#_ENREF_1), [4](#_ENREF_4), [7](#_ENREF_7), [8](#_ENREF_8)]. New infection also is more likely to directly progress to active disease in HIV-positive individuals and self-cure from active infection does not occur [[9](#_ENREF_9)].

Upon development of active TB, patients are immediately considered to be infectious if they develop pulmonary TB (PTB) and have an increased mortality risk due to TB. Children with PTB are considered to be less infectious than adults (by a factor of 1/5). In children 85% develop TB that is difficult to diagnose with current widely available diagnostic methods [[10-13](#_ENREF_10)]. In adults the development of EPTB and sputum scarce TB will depend on the HIV-status of the individual [[14-16](#_ENREF_14)] (Table E1).

Some patients will not have access to health care and diagnostics for TB and will remain infectious until they either self-cure or die (Table E1). Other patients will get diagnosed and will exit the subpopulation of active diagnosed cases at a rate defined as the inverse of the mean time to initial diagnosis. The likelihood of being diagnosed and the time to initial diagnosis will depend on the diagnostic method available and the HIV status. Individuals with active TB are assumed to undergo diagnostic attempts at a defined rate.

Unlike other models that assume diagnostic attempts to reflect tests with a single diagnostic or defined series of diagnostic tests, our conceptualization of “diagnostic attempt” is more inclusive and incorporates all initial and follow-on testing that is performed until a diagnosis of TB is either made or excluded by the diagnosing practitioner or team of practitioners [[17](#_ENREF_17), [18](#_ENREF_18)]. One diagnostic attempt may include clinical judgment, radiography and other tests in addition to the diagnostic test for TB specifically (smear microscopy or molecular test) and is considered to lead to a diagnosis (Table E1). By using this more inclusive definition of “diagnostic attempt,” we maximize our ability to account for empiric treatment but may underestimate the impact of a rapid diagnostic test in terms of reducing diagnostic delays, which are intrinsically incorporated into our rate of diagnostic attempt. Extrapulmonary TB requires an invasive sample for microbiological proof, thus diagnostic attempts are often delayed (diagnostic rate is reduced by half compared to PTB) [[19-23](#_ENREF_19)].

At the time of diagnosis, we assume that 85% of patients obtain treatment [[24](#_ENREF_24)]. Patients with active TB who receive treatment are instantaneously placed into one of three subpopulations:

1. Cured/Recovered: Those who are cured from TB whether or not that completed a full course of therapy.
2. Active, previously treated TB: Those who default or complete therapy but will relapse.
3. Failure: Those who fail therapy.

Depending on the baseline susceptibility of the strain (e.g. INH-resistant) the patients may also develop further resistance. Patients who develop additional resistance mutations are assigned directly to the failing group in the respective drug-resistant compartment (e.g. MDR resistance). The distribution into the subpopulation compartments (cured, previously treated active TB, failure and resistance) reflects the percentages as reported in the literature (Table E2).

We presumed that, before year zero, drug-susceptibility testing (DST) is limited only to those who have failed a previous course of TB therapy and remain symptomatic [[25](#_ENREF_25)]. In all other cases, patients are treated with standard short-course (first-line) therapy. If resistance is present at initial diagnosis, a higher proportion of patients will fail, recur or develop further resistance (Table E2). Patients who already failed therapy are assumed to receive second-line therapy for MDR-TB after the duration that it takes for them to be identified as failing first-line therapy (six to eight months).

Patients with recurrent TB after completing an initial course of therapy are assumed to be diagnosed at the same rate as new cases. The likelihood of being diagnosed depends on the diagnostic method available [[25](#_ENREF_25)]. If the patient does not receive DST or the DST does not diagnose resistance, treatment including an aminoglycoside and lasting a total of eight months (“category II”) is assumed. In contrast, if the patient is diagnosed with a resistance mutation based on DST, a second-line regimen is assumed, with correspondingly higher cure rates (Table E2). Patients who fail therapy are assumed to be re-diagnosed at twice the rate of new cases.

All patients with active PTB are considered infectious. Patients who are failing but on partially active therapy (i.e. 1 or 2 active drugs) are assumed to be as infectious as smear-negative patients, who are responsible for about 20% of cases in contact and outbreak investigations [[26](#_ENREF_26)]. Similarly, children are presumed to be less infectious overall (presumed to be similar to failure cases), with likely no infectivity at all in children under 5, although data is very limited.

Active regimens immediately render the patient non-infectious and return the patient’s mortality risk to that of an uninfected individual [[27](#_ENREF_27)]. Patients who are cured may get reinfected but are considered to have partial protection against reinfection similar to that of latent TB infection [[1](#_ENREF_1)]. If these patients acquire infection again, they progress to the previously-treated active TB group.

**Table E1: Parameter estimates**

|  | **Value** | **Range** | **Reference** |
| --- | --- | --- | --- |
| Non-TB death rate per year in adults (life expectancy 60 years) | 0.022 | 0.02-0.025 |  |
| Non-TB death rate per year in children | 0.0003 | 0.0001-0.0005 |  |
| TB mortality per year | 0.15 | 0.10–0.22 | [[25](#_ENREF_25)] |
| TB mortality per year in HIV co-infected | 0.50 | 0.4-0.7 | [[6](#_ENREF_6)] |
| HIV related mortality per year | 0.05 | 0.03-0.1 | [[5](#_ENREF_5)] |
| HIV prevalence | 0.003 | 0.002-0.005 | [[28](#_ENREF_28)] |
| Attenuation of infectiousness by resistance mutation before study starts   - INH - MDR - XDR | 0.988 0.857  0.5 | 0.9-1.0  0.6-0.9  0.4-0.7 | [[29-33](#_ENREF_29)] |
| Attenuation of infectiousness by HIV status | 0.5 | 0.3-0.8 | [[29-34](#_ENREF_29)]([29-34](#_ENREF_29)) |
| Relative infectivity of cases failing therapy and children with TB | 0.2 | 0.16–0.28 | [[26](#_ENREF_26), [35](#_ENREF_35)] |
| Relative infectivity of patients with TB and HIV co-infection | 0.5 | 0.3-0.7 | [[36](#_ENREF_36)] |
| Proportion that develops EPTB or PTB that cannot be diagnosed on sputum in HIV negative   - Adults - Children (weighted average among different age groups) | 0.18  0.85 | 0.15-0.25  0.6-0.9 | [[10](#_ENREF_10), [11](#_ENREF_11), [13](#_ENREF_13), [37](#_ENREF_37), [38](#_ENREF_38)] |
| Proportion that develops EPTB or PTB that cannot be diagnosed on sputum in HIV positive   - Adults - Children (weighted average among different age groups) | 0.35  0.85 | 0.3-0.7  0.6-0.9 | [[10](#_ENREF_10), [13-16](#_ENREF_13), [39](#_ENREF_39)] |
| Relative protection from reinfection in latent/recovered TB in   - HIV negative - HIV positive | 0.45  0 | 0.4-0.55  0-0.2 | [[1](#_ENREF_1), [3](#_ENREF_3), [4](#_ENREF_4)] |
| Proportion of TB infections progressing rapidly to active TB in   - HIV negative - HIV positive | 0.14  0.25 | 0.05–0.14  0.16-0.27 | [[2](#_ENREF_2), [9](#_ENREF_9)] |
| Endogenous reactivation rate per year in   - HIV negative - HIV positive | 0.0005  0.05 | 0.08–1.4 x10^-3^  0.03–0.05 | [[7](#_ENREF_7), [8](#_ENREF_8), [40](#_ENREF_40)] |
| Rate of self-cure in active TB per year   - HIV negative - HIV positive | 0.1  0 | 0.08-0.28  0-0.2 | [[41](#_ENREF_41), [42](#_ENREF_42)] |
| Percent of incident cases without access to diagnosis | 15 | 5-25 | [[43](#_ENREF_43), [44](#_ENREF_44)] |
| Sensitivity of current diagnostic standard per diagnostic attempt in HIV negative for   - PTB - TB difficult to diagnose (EPTB, sputum scarce) | 0.80  0.60 | 0.6-0.9  0.2-0.7 | [[25](#_ENREF_25), [45-51](#_ENREF_45)] |
| Sensitivity of molecular methods per diagnostic attempt for PTB in HIV negative | 0.95 | 0.75-0.98 | [[22](#_ENREF_22), [52-56](#_ENREF_52)] |
| Proportional sensitivity of current diagnostics standard and molecular methods in HIV-positive compared to HIV-negative | 0.8 | 0.6-0.9 | [[22](#_ENREF_22), [25](#_ENREF_25), [45-47](#_ENREF_45), [52](#_ENREF_52), [57](#_ENREF_57)] |
| Sensitivity of molecular methods for INH resistance detecting katG (high-level resistance) and inhA (low-level resistance) | 0.88 | 0.7-0.95 | [[58](#_ENREF_58)] |
| Sensitivity of molecular methods for RIF resistance (as a marker of MDR) | 0.94 | 0.90-0.96 | [[22](#_ENREF_22), [57](#_ENREF_57)] |
| Sensitivity of molecular methods for FQ and AG resistance (i.e. XDR) | 0.84 | 0.60-0. 90 | [[59-61](#_ENREF_59)] |
| Sensitivity of phenotypic culture-based methods for RIF, INH, FQ and AG resistance | 1 |  | Assumed |
| Duration of illness before diagnostic attempt completed with standard test (months) for new and relapse cases | 8 | 4-12 | [[19-22](#_ENREF_19)] |
| Duration of illness before diagnostic attempt completed with molecular test (months) for new and relapse cases | 6 | 4-12 | [[19-22](#_ENREF_19)] |
| Proportional decrease in diagnostic rate for patients with EPTB if sampling of the site of disease is necessary for diagnostic test | 0.5 | 0.5-1 | [[19-23](#_ENREF_19)] |
| Proportional increase in diagnostic rate for patients failing therapy | 2 |  | [[19-22](#_ENREF_19)] |
| Proportional increase in diagnostic rate in patients with HIV | 2 |  | [[21](#_ENREF_21)] |
| Proportion of patients that starts therapy after a TB diagnosis was achieved | 0.85 | 0.81-0.89 | [[62](#_ENREF_62), [63](#_ENREF_63)] |

**Table E2: Estimates of treatment success rates**

| Patients with new infection on standard short-course therapy | | | |
| --- | --- | --- | --- |
| Patients with sensitive TB. Proportions:  Cured  Recurrence (default + relapse)  Failing  Developing INH resistance  Developing MDR resistance | 0.88  0.09  0.025  0.004  0.001 | 0.75-0.95  0.02-0.1  0.01-0.03  0.003-0.01  0.0005-0.03 | [[25](#_ENREF_25), [64-66](#_ENREF_64)] |
| Patients with INH- monoresistant TB treated with standard short-course therapy (DST not done). Proportions:  Cured  Recurrence (default + relapse)  Failing (not due to new drug resistance)  Developing MDR resistance | 0.80  0.09  0.10  0.01 | 0.65-0.90  0.05-0.2  0.03-0.2  0.001-0.02 | [[25](#_ENREF_25), [65-70](#_ENREF_65)] |
| Patients with MDR TB treated with standard short-course therapy (DST not done). Proportions:  Cured  Recurrence (default + relapse)  Failing (not due to new drug resistance)  Developing XDR resistance | 0.25  0.35  0.35  0.05 | 0.2-0.4  0.10-0.50  0.3-0.70  0.05-0.1 | [[25](#_ENREF_25), [67](#_ENREF_67), [68](#_ENREF_68), [71-73](#_ENREF_71)] |
| Patients with XDR TB treated with standard short-course therapy (DST not done). Proportions:  Cured  Recurrence (default + relapse)  Failing | 0.15  0.40  0.45 | 0.05-0.3  0.10-0.60  0.4-0.70 | Estimate |
| Patients with new infection on therapy based on DST | | | |
| Patients with INH-monoresistant TB on active therapy based on DST. Proportions:  Cured  Recurrence (default + relapse)  Failing (not due to new drug resistance)  Developing MDR resistance | 0.88  0.09  0.029  0.001 | 0.75-0.95  0.05-0.17  0.02-0.11  0.001-0.005 | [[25](#_ENREF_25), [65](#_ENREF_65), [70](#_ENREF_70), [74-77](#_ENREF_74)] |
| Patients with MDR TB on active therapy based on DST. Proportions:  Cured  Recurrence (default + relapse)  Failing  Developing XDR resistance | 0.52  0.23  0.176  0.069 | 0.40-0.83  0.15-0.35  0.1-0.30  0.03-0.1 | [[25](#_ENREF_25), [73](#_ENREF_73), [78-85](#_ENREF_78)] |
| Patients with XDR TB on active therapy based on DST. Proportions:  Cured  Recurrence (default + relapse)  Failing | 0.35  0.33  0.32 | 0.2-0.5  0.2-0.4  0.2-0.4 | [[25](#_ENREF_25), [82](#_ENREF_82), [86-89](#_ENREF_86)] |

**Table E3: Model compartments**

All departments are subdivided by age, pulmonary versus extrapulmonary TB, drug-susceptibility and HIV-status. In total 164 compartments.

| **Compartment** | **Description** |
| --- | --- |
| *S_a,h_* | Susceptible never infected before  Maximum risk of TB infection |
| *L_d,a,h_* | Latently infected  Offers partial protection against re-infection |
| *A_d,a,h,t_* | Actively infected that will be diagnosed and treated  Infectious, increased mortality |
| *N_d,a,h,t_* | Actively infected but never diagnosed  Infectious, increased mortality |
| *F_d,a,h,t_* | Failure – requiring ongoing therapy  Individuals who develop resistance directly go from active treatment into the respective failed resistant compartment  Infectious at the rate of smear –negative cases |
| *R_d,a,h,t_* | Active recurring TB – Individuals who have active infection because they default, relapse or reinfection |
| *C_d,a,h_* | Cured/Recovered  At risk for recurrent infection with partial infection conferred by prior infection |

Legend: *d* refers to drug susceptibility (sensitive (s), multidrug-resistant (MDR), extensively drug-resistant (XDR) or INH-resistant (INH); t=type of infection (PTB, EPTB), h=HIV status (positive, negative), a=age group (children, adults)

1. **Description of parameters**

This section provides a more detailed description of the primary parameters for which the most data exist to inform parameter estimates. The estimates for parameters with ranges and citations are listed in Table E1.

The population size of the hypothetical model population is set at 100,000. Individuals enter the model at birth, being HIV-negative and uninfected with TB. They exit the model upon dying or reaching their 60th birthday. Mortality rates depend on an individual’s TB and HIV status. Patients with active TB have an increase in the mortality rate of 0.15/year for HIV negative and 0.5/year for HIV positive (average for both smear negative and smear positive; incorporating an early, subclinical phase) over the baseline mortality of the uninfected. Patients with HIV-infection only (no TB infection) have a mortality rate that is increased by 0.05/year over the mortality rate of the uninfected. Patients who are partially treated (i.e. only 1 or 2 active drugs) are considered to have the same mortality rate than patients who have smear-negative TB (25% of smear-positive TB). Adult HIV-prevalence was set at the numbers reported for India in the United Nations report [[5](#_ENREF_5)]. We estimate an annual risk of HIV infection based on the prevalence of 0.001.

The transmission rate (β) denotes the number of secondary infections per infectious case. We calculate the transmission based on the TB incidence in India in 2011 (181/100,000) [[25](#_ENREF_25)].

Assuming an increase in resistance since introduction of anti-mycobacterial therapy in the 1950s, an attenuation of infectivity has to be expected for MDR strains to explain the currently observed MDR estimates. Similar results have also been shown in laboratory experiments [[31-33](#_ENREF_31)]. Laboratory experiments on the transmissibility of INH-monoresistant TB suggest less attenuation (range from 0.7 to 1.1) [[29-31](#_ENREF_29), [90](#_ENREF_90)]. In our model we calculated the attenuation necessary to reproduce a constant increase in resistance since the 1950s. However, this proved analytically impossible for INH-monoresistant TB without making unreasonable assumptions (e.g., more transmissible than wild-type TB, very poor treatment outcomes).

Thus, we instead calibrated the transmission rate of INH-monoresistant TB to provide a steady-state level of INH-monoresistance (at 15% of new cases) over the past 60 years. This is consistent with data of high INH-monoresistance from early surveillance reports and the lack of a significant increase in INH-resistance in India since that time [[25](#_ENREF_25), [91-93](#_ENREF_91)]. This procedure required only a minimal decrease in the transmission fitness of INH-monoresistant. After initiating this steady state, we calibrated the relative infectiousness of MDR-TB and XDR-TB such that the modeled incidence among new (not previously treated) cases was 2.1% and 0.2%, as estimated in India in 2011 respectively [[25](#_ENREF_25)]. However, given the possibility of compensatory mutations that restore the transmissibility, we do a sensitivity analysis around the attenuation parameters.

The proportion of TB infections that progresses rapidly to active TB is taken as the proportion of patients who develop active TB within one year of TB infection from Vynnycky and Fine’s estimation in a British Population [[2](#_ENREF_2)]. Of note, this estimate of 14% is greater than the classically assumed 5%, or half of a 10% lifetime risk for active TB if infected in childhood. Vynnycky and Fine suggest that the risk of rapid progression is higher in adults (14%) than in children (4%). To account for the possibility of overestimating this parameter, we perform a univariate sensitivity analysis to a lower bound of 5%.

The percentage of patients who are never diagnosed due lack of access to care also is a matter of debate. Data exists from hospital studies primarily in an HIV-positive population where up to three fourth of patients die of TB and a quarter was never suspected to have TB prior to dying [[43](#_ENREF_43)]. The proportion might be even higher in patients dying in the community but studies are limited [[44](#_ENREF_44)]. However, these estimates do not take into account self-cure and estimates are certainly presumed to be lower in HIV-negative patients [[25](#_ENREF_25)]. A sensitivity analysis was done to a lower limit of 5% and an upper limit of 25% to account for uncertainty in this parameter value.

The annual endogenous reactivation rate in HIV-negative patients is taken from Ferebee’s 1970 review of TB chemoprophylaxis trials [[40](#_ENREF_40)]. The estimate of the percent of patients that self cure is taken from prior work of Enarson and Rouillon [[94](#_ENREF_94)].

The diagnostic rate is calculated as the inverse of the mean time to initial diagnosis, which is the sum of the disease duration of untreated TB and the provider delay after presentation. The mean time to diagnosis varies between studies [[19-21](#_ENREF_19)]. Given that the estimate may affect the calculated TB incidence significantly, we perform a sensitivity analysis to account for a range of duration until diagnosis. The delay in diagnosing EPTB is even more substantial, likely because of the lack of suspicion for the diagnosis and the difficulty in obtaining a sample for diagnosis [[23](#_ENREF_23)]. In contrast, diagnosis in HIV-patients is more actively pursued as patients already have access to the health care system and the need for diagnosing co-infection to prevent morbidity and mortality is recognized [[21](#_ENREF_21), [95](#_ENREF_95)]. Thus, we assume that diagnostic attempts happen on average twice as often for HIV-positive individuals than for HIV-negative individuals. At the time of diagnosis, we assume that 85% of patients obtain treatment [[24](#_ENREF_24)].

The sensitivity of TB detection with established methods can be estimated from case detection rates in the recent WHO report [[25](#_ENREF_25)]. For the Xpert MTB/RIF accuracy estimates have been published in demonstration studies and a recent meta-analysis by Steingart et al. [[22](#_ENREF_22), [57](#_ENREF_57), [96](#_ENREF_96)]. The accuracy of molecular testing for rifampin for Xpert has also been well described in the initial implementation studies [[22](#_ENREF_22), [57](#_ENREF_57)]. We also assumed that a novel highly deployable test is most likely an antigen-based test, and would not, at least in its first iteration, contain the capacity for drug susceptibility testing.

Treatment success estimates are taken from the most recent WHO Global TB control report and other publications as outlined in the table.

We conducted uni-variate sensitivity analyses where one parameter is varied (across the ranges specified in Table E4) and the others parameters held constant. Furthermore, to estimate variability associated with simultaneous changes in all parameters, we also conducted a probabilistic uncertainty analysis, using Latin Hypercube Sampling to select values randomly from beta distributions (for parameters, e.g. probabilities, bounded from 0 to 1) or gamma distributions (for parameters, e.g. rates, bounded from 0 to infinity) for each parameter across a range of 25% unless otherwise indicated. Simulations that caused a two-fold increase or 50% decrease in TB incidence over 10 years were rejected. We conducted more than 10,000 independent simulations in this fashion, thus generating 95% uncertainty ranges, defined as the intervals bounded by the 2.5 and 97.5 percentiles of all acceptable simulations.

**Table E4: Univariate sensitivity analysis – base-case value and range**

| **Parameter** | **Value** | **Range** |
| --- | --- | --- |
| Non-TB death rate per year (life expectancy 60 years) in adults | 0.022 | 0.017-0.028 |
| TB mortality per year | 0.15 | 0.11-0.19 |
| TB mortality per year in HIV co-infected | 0.5 | 0.4-0.6 |
| Attenuation of infectiousness by INH resistance mutation | 0. 988 | 0.98-1.0 |
| Attenuation of infectiousness by MDR resistance mutation | 0.86 | 0.7-1.0 |
| Attenuation of infectiousness by XDR resistance mutation | 0.5 | 0.4-1.0 |
| HIV incidence per year | 0.001 | 0.0007-0.0013 |
| Proportion that develops EPTB or PTB that cannot be diagnosed on sputum in HIV negative   - Adults - Children (weighted average among different age groups) | 0.18  0.85 | 0.14-0.23  0.6-0.90 |
| Relative protection from reinfection in latent/recovered TB in HIV negative | 0.45 | 0.4-0.5 |
| Relative protection from reinfection in latent/recovered TB in HIV positive | 0 | 0-0.2 |
| Proportion of TB infections progressing rapidly to active TB in HIV negative | 0.14 | 0.05-0.14 |
| Proportion of TB infections progressing rapidly to active TB in HIV positive | 0.25 | 0.16-0.27 |
| Endogenous reactivation rate per year in HIV negative | 0.0005 | 0.08-1.4x10^-3^ |
| Endogenous reactivation rate per year in HIV positive | 0.05 | 0.03-0.06 |
| Rate of self-cure in active TB per year in HIV negative | 0.1 | 0.08-0.2 |
| Rate of self-cure in active TB per year in HIV positive | 0 | 0-0.2 |
| Proportion of patients without access to diagnostics | 0.1 | 0.05-0.25 |
| Sensitivity of current diagnostic standard per diagnostic attempt for PTB | 0.80 | 0.6-0.9 |
| Sensitivity of current diagnostic standard per diagnostic attempt for extrapulmonary TB | 0.60 | 0.4-0.8 |
| Sensitivity of molecular methods per diagnostic attempt | 0.95 | 0.8-0.98 |
| Sensitivity of molecular methods for RIF resistance detecting | 0.94 | 0.9-0.96 |
| Sensitivity of molecular methods for INH resistance detecting katG (high-level resistance) and inhA (low-level resistance) | 0.88 | 0.75-0.95 |
| Sensitivity of molecular methods for resistance detecting fluoroquinolone and aminoglycoside resistance | 0.84 | 0.6-0.90 |
| Duration of illness before diagnostic attempt completed (months) for new and relapse cases | 6 | 4-8 |
| Duration of failing therapy before diagnostic attempt completed (months) | 3 | 2-6 |

1. **Model parameters and there symbolic representation**

**Table E5: Model parameters and their symbolic representation**

| **Parameter** | **** |
| --- | --- |
| Transmission rate (transmission events per infectious person-year; subscript *d* indicates drug-susceptibility) | *β_δ_* |
| Attenuation of infectiousness by resistance (*d),* HIV status (*h*), age group (*a*) and type of disease (*t*) (indicated by subscript)   - XDR - MDR - INH - HIV positive - Children - EPTB | *c_d,h,a,t_* |
| Force of infection (with subscript indicating drug-susceptibility *d*, HIV status *h*, age group *a*, and type of infection *t)* | *λ_d,h,a,t_* |
| Endogenous reactivation rate, per year | *ε_η_* |
| Proportion of infections progressing rapidly to active TB | *π_η_* |
| Relative protection from reinfection in latent/recovered TB | *_η_* |
| TB mortality rate, per year (subscript *h* indicates HIV status) | *_h_* |
| Baseline mortality rate (subscript *a* indicates age groups; subscript *h* indicates HIV status), per year | *μ_α,h_* |
| Spontaneous cure rate, per year | *_η_* |
| Relative transmission rate (per year), failing cases | *σ* |
| Duration of illness before diagnostic attempt completed (subscript *t* indicates type of disease; subscript *h* indicates HIV status) | *δNR_t,h_* |
| Duration of failing therapy before diagnostic attempt completed with molecular test (months) | *δF_t,h_* |
| Diagnostic rate for new or default/relapse or reinfection cases | *τNR_t,h_* |
| Diagnostic rate for failure cases | *_t,hλ_* |
| Proportion of patients without access to diagnostics (independent of age, HIV status or drug-susceptibility status) | *η* |
| Probability of receiving a molecular diagnostic test as a new case | *ω__* |
| Probability of receiving a molecular diagnostic test as a retreatment case | *ω_re_* |
| Probability of receiving a molecular diagnostic test when failing therapy | *ω_fail_* |
| Proportion of patients initiating treatment after diagnosis (independent of age, HIV status or drug-susceptibility status) | *κ* |
| Probability of cure, default/relapse, failure, INH, MDR or XDR resistance development (subgroup defined by disease status: cured=c, default/relapse=def, failure=fail, INH, MDR, or XDR resistant = INH, MDR or XDR) in new active cases | *δ* |
| Probability of cure, default/relapse, failure, INH, MDR or XDR resistance development (subgroup defined by disease status: cured=c, default/relapse=def, failure=fail, INH, MDR, or XDR resistant = INH, MDR or XDR) in active retreatment cases | *E* |
| Probability of cure, default/relapse, failure, INH, MDR or XDR resistance development (subgroup defined by disease status: cured=c, default/relapse=def, failure=fail, INH, MDR, or XDR resistant = INH, MDR or XDR) in failure cases | *δFail* |

**Secondary parameters**

1. Transmission rate for resistant strains:

The transmission rate for resistant strains is a function of the attenuation of the individual strains and the transmission rate (β_δ_. The transmission rate varies by resistance strain, HIV status, age-group and disease types with different levels of attenuation (c_d,h,a,t_).

INH: *β_INH_* = *β*  *c_INH_*

MDR: *β__* = *β*  *c_MDR_*

XDR: *β_X_* = *β*  *c_XDR_*

1. Diagnostic and treatment rate:

The diagnostic rate is defined as the inverse of the mean time to initial diagnosis. The time to initial diagnosis depends on the case category of the patient (failing versus new/relapse) and the diagnostic test the patient receives. Failing cases are in the system already also probably have more pronounced symptoms and are therefore more likely to be diagnosed faster. The time to diagnosis for new and relapse cases incorporates a subclinical period where the patient is infectious but not seeking care yet. Once diagnosed only a proportion of patients ) actually initiates treatment while others are lost to follow up (independent of age, HIV status or drug-susceptibility status).

 active * *NR = 1/*δ*NR* 

Active, previously treated cases: *τ*NR = 1/*δ* ^NR^* 

Failure  *τ*F= 1/*δ*F* 

1. Force of Infection (λ)

TB infection is modeled as a density-dependent process, a function of the transmission rate (β; attenuated if the source case is a resistant case; subscript d indicates drug susceptibility), age and HIV-status (decreased infectiousness of children and HIV-positive patients), number of individuals with infectious TB (*A_d_*, active new cases; *R_d_*, active, previously treated cases; *F_d_*, individuals failing therapy), divided by the total size of the population. Failure cases are also presumed to have an attenuated infectivity on the level of a smear-negative case due to partial treatment ( .

λ_d,h,a,t_(t)= β * c*_d,a,h,t_* * c_HIV_ * (A_d,h,a,t_(t)+ R_d,h,a,t_(t)+ N_d,h,a,t_(t)+  * F_d,h,a,t_(t)) /

(S_d,h,a,t_(t) + L_d,h,a,t_(t) + N_d,h,a,t_(t) + A_d,h,a,t_(t) + F_d,h,a,t_(t) + C_d,h,a,t_(t) + R_d,h,a,t_(t))

d) Total mortality (mort)

Totally mortality is a sum of baseline mortality by age group, HIV mortality and TB mortality multiplied by the respective compartment.

mort(t) = μ*_a,h_** (S*_d,h,a,t_*(t) + L*_d,h,a,t_*(t) + A*_d,h,a,t_*(t) + F*_d,h,a,t_*(t) + C*_d,h,a,t_*(t) + R*_d,h,a,t_*(t)) +

μTB*_h_** (A*_d,h,a,t_*(t) + R*_d,h,a,t_*(t)) + N*_d,h,a,t_* (t) + 0.25* F*_d,0,a,t_*(t))

1. **Model Equations**

In the following equations, the compartmental subpopulations are denoted by capital letters. All populations are represented by single letters. Populations without active TB are susceptible (*S*), latently infected (*L*), or cured/recovered (*C*) status. Populations with active TB are active, new cases with access to diagnosis (*A*) or active, new cases without access to diagnosis (*N*) status, failure cases (F) and active, previously treated cases (R). Subscript *h* refers to HIV status (0 = uninfected, 1 = infected), *d* refers to drug susceptibility (sensitive =0, INH-monoresistant =1, multidrug-resistant (MDR)=2, or, extensively-resistant (MDR)=3), *a* refers to age group (0 = children, 1 = adults) and *t* refers to type of disease (0=PTB, 1=EPTB). Time-dependent parameters are followed by (t). Rates of flow between compartments are governed by the system of ordinary differential equations listed in equations 2-6. The model is programmed in Python, and the source code for the model is available from the first author on request.

Equation 1. Susceptible Compartments (S)

dS*_h,a_*(t)/dt = mort(t) ‒ (λ_d,h,a,t_(t) * S*_h,a_*(t) + μ*_a,h_**S*_h,a_*(t))

where mort(t) is the sum of all mortality, λ_d,h,a,t_(t) is the force of infection for all different types of TB (drug-susceptible, MDR, INH-resistant), μ*_a_* is the non-TB mortality rate (dependent on *a* age group), and μHIV*_h_* is the HIV-related mortality rate.

Thus, uninfected individuals leave this compartment through infection and death, and the compartment is replenished at a rate that matches total mortality. These compartments are subdivided only by HIV-status and age group.

Equation 2. Latently Infected Compartments (L)

dL*_d,h,a_*(t)/dt = [λ_d,h,a,t_(t) * (1 ‒ π_h_) * S_h,a_(t)

+ λ_d,h,a,t_(t) * (1 ‒ π_h_) * (1 - _h_ L*_d,h,a_*(t)

+ _h_  *_d,h,a,t_*(t)  _ _ *_d,h,a,t_*(t)]* *

- λ_d,h,a,t_(t) * (1 ‒ ι_h_) * L*_d,h,a_*(t)

- [ε_h_ + μ*_a,h_*] * L*_d,h,a_*(t)

where λ_d,h,a,t_(t) is the force of infection for all different types of TB and π_h_ is the proportion of recent infections that progress rapidly to active TB, ι_h_ is the relative protection from reinfection in latent/recovered TB, _h_ is the rate of self-cure, ε_h_ is the endogenous reactivation rate, and μ_a,h_ is the non-TB mortality rate.

Thus, susceptible individuals who get newly infected or latently infected patients who get infected with a different strain but do not progress rapidly to active disease, as well as patients who self-cure make up these latent compartments. Latently-infected individuals leave these compartment through TB reinfection with a different strain than the primary strain (with rapid progression they go into respective active compartments; without rapid progression they go into the respective latent compartments), endogenous reactivation, and death. These compartments are subdivided by drug-susceptibility, HIV-status and age group.

Equation 3. Active TB Compartment (A)

dA*_d,h,a_*_,t_(t)/dt = λ_d,h,a,t_(t) * π_h_ * (1 – ) * S_h,a_(t)

+ λ_d,h,a,t_(t) * π_h_ * (1 - _h_ * (1 – )  L_d, h, a_(t)

+ ε_h_ * (1 – )  L_d,h,a,_(t)

- *τNR_t,h_* * δ* A_d,h,a,t_(t)

- [υ_h_ + μ_a,h_ + μTB_h_] * A_d,h,a,t_(t)

where λ_d,h,a,t_(t) is the force of infection for all different forms of TB, π_h_ is the proportion of recent infections that progress rapidly to active TB, ι_h_ is the is the relative protection from reinfection in latent/recovered TB,  is the proportion without access to diagnostics, ε_h_ is the endogenous reactivation rate, *τNR_t,h_* is the diagnostic rate for new infections, δ is the probability of cure (δ_χ_), default/relapse(δ_def_), failure (δ_fail_) or resistance development (δ_INH_, δ_MDR_) with diagnosis and treatment in new cases, _h_ is the rate of self-cure, μ_a,h_ is the non-TB mortality rate, and μTB_h_ is the TB mortality rate.

Thus, susceptible individuals and latently infected individuals (those not protected through prior infection) who progress rapidly into active infection, as well as those who reactivate constitute the active diagnosed compartments. Individuals leave the compartment through diagnosis at a defined diagnostic rate and treatment resulting in cure, default/relapse, failure or development of resistance, spontaneous cure, or death (from TB or other causes). Active compartments are subdivided by drug-susceptibility, HIV-status, type of disease and age group.

Equation 4. Never-diagnosed, active compartment (N)

dN*_d,h,a_*_,t_(t)/dt = λ_d,h,a,t_(t) * π_h_ *  * S_h,a_(t)

+ λ_d,h,a,t_(t) * π_h_ * (1 - _h_ *   L_d,a,h_(t)

+ ε_h_*   L_d,h,a,_(t)

- [υ_h_ + μ_a,h_ + μTB_h_] * N_d,h,a,t_(t)

where λ_d,h,a,t_(t) is the force of infection for all different forms of TB, π_h_ is the proportion of recent infections that progress rapidly to active TB, ι_h_ is the is the relative protection from reinfection in latent/recovered TB,  is the proportion without access to diagnostics, ε_h_ is the endogenous reactivation rate, _h_ is the rate of self-cure, μ_a,h_ is the non-TB mortality rate, and μTB_h_ is the TB mortality rate.

Thus, susceptible individuals and latently infected individuals (those not protected through prior infection) who progress rapidly into active infection as well as those who reactivate and never get diagnosed due to lack of access to diagnostics constitute the active never-diagnosed compartment. Individuals leave the compartment only through spontaneous cure, or death (from TB or other causes). Similar to the active, diagnosed compartments, these active, never-diagnosed compartments are subdivided by drug-susceptibility, HIV-status, type of disease and age group.

Equation 5: Active, previously treated cases (R)

dR*_d,h,a_*_,t_(t)/dt = *τNR_t,h_* * δ_def_ * A_d,h,a,t_(t)

+ *τF_t,h_* * δFail_def_ * F_d,h,a,t_(t)

+*τNR_t,h_* * δRe_def_ * R_d,h,a,t_(t)

+ λ_d,h,a,t_(t) * π_h_ * (1 - _h_ * _d,a,h,t_

-*τNR_t,h_* * δRe

- [υ_h_ + μ_a,h_ + μTB_h_] * R_d,h,a,t_(t)

where *τNR_t,h_* is the diagnostic rate for new infections, δ_def_ is the probability of default/relapse in new cases, *τF_t,h_* is the diagnostic and treatment rate for individuals failing therapy, δFail_def_ is the probability of default/relapse in failing cases, δRe_def_ is the probability of default/relapse in active, previously treated cases,λ_d,h,a,t_(t) is the force of infection, π_h_ is the proportion of recent infections that progress rapidly to active TB, ι_h_ is the is the relative protection from reinfection in latent/recovered TB, δRe is the probability of cure, default/relapse, failure or resistance development with diagnosis and treatment in active, previously treated cases, _h_ is the rate of self-cure, μ_a,h_ is the non-TB mortality rate, and μTB_h_ is the TB mortality rate.

Thus, individuals enter the compartment through relapse or default out of the active new (A_d,h,a,t_), active previously treated (R_d,h,a,t_) or failure (F_d,h,a,t_) compartments or through reinfection of patients who had achieved cure from a prior infection (C_d,h,a,t_). Individuals leave the compartment through diagnosis at a defined diagnostic rate for retreatment cases and resulting in treatment and cure, default/relapse, failure or development of resistance. Furthermore, they can leave the department through self-cure, or death (from TB or other causes). Similar to the active, new compartments, these active, previously treated compartments are subdivided by drug-susceptibility, HIV-status, type of disease and age group.

Equation 6: Failure (F)

dF*_d,h,a_*_,t_[t]/dt = *τNR_t,h_* * δ_fail_ * A_d,h,a,t_(t)

+ *τNR_t,h_* * δ_INH,MDR,XDR_ * A_d,h,a,t_(t)

+ *τNR_t,h_* * δRe_fail_ * R_d,h,a,t_(t)

+ *τF_t,h_* * δFail_INH/MDR/XDR_ * F_d,h,a,t_(t)

- *τF_t,h_* * δFail

- [μ_a,h_+ 0.25*μTB_h_] * F_d,h,a,t_(t)

where *τNR_t,h_* is the diagnostic and treatment rate for new infections, δ_fail_ and δRe_fail_ are the probability of failure in active new and previously treated cases, δ_INH,MDR,XDR_ is the probability of failure and development of resistance (INH monoresistance, MDR or XDR) with first line therapy (either standard or based on drug-susceptibility testing) out of an active compartment, δFail_INH/MDR/XDR_ is the probability of failure and development of resistance (INH monoresistance, MDR or XDR) with standard category II treatment or treatment guided by drug-susceptibility in failing cases, which results in a change from one failure compartment into another (determined by acquired drug-resistance). *τF_t,h_* is the diagnostic and treatment rate for individuals failing therapy, δFail is the probability of cure, default/relapse, failure or resistance development with diagnosis and treatment in failure cases, and μ_a,h_ is the non-TB mortality rate and μTB_h_ is the TB mortality rate (multiplied by 0.25 as failure cases are considered partially treated).

Thus, individuals enter the compartment through failing therapy for a new infection or failing retreatment for new infection after having been previously treated for TB or after default or relapse (A_d,h,a,t_ and R_d,h,a,t_). Individuals leave the compartment through diagnosis at a defined diagnostic rate and treatment resulting in cure, default/relapse, failure or development of resistance, or death (from other causes). Similar to the active, new compartments, failure compartments are subdivided by drug-susceptibility, HIV-status, type of disease and age group.

Equation 7: Recovered/Cured Compartment (C)

dC*_d,h,a_*[t]/dt = *τNR_t,h_* * δ_c_* A_d,h,a,t_(t)

+ *τNR_t,h_* * δRe_c_ * R_d,h,a,t_(t)

+ *τF_t,h_* * δFail_c_ * F_d,h,a,t_(t)

+ λ_d,h,a,t_(t) * (1 - π_h_) * (1 - _h_ * _d,h,a_(t)

+ υ_h_ * R_d,h,a,t_(t)

- λ_d,h,a,t_(t) * (1 - _h_ * C_d,h,a_(t)

- μ_a,h_* C_d,h,a_(t)

where *τNR_t,h_* is the diagnostic and treatment rate for new infections, δ_c_ is the probability of cure in new cases, *τF_t,h_* is the diagnostic and treatment rate for individuals failing therapy, δFail_c_ is the probability of cure in failing cases, δRe_c_ is the probability of cure in active, previously treated cases,λ_d,h,a,t_(t) is the force of infection for all TB, π_h_ is the proportion of recent infections that progress rapidly to active TB, ι_h_ is the relative protection from reinfection in latent/recovered TB, _h_ is the rate of self-cure and μ_a,h_ is the non-TB mortality rate.

Thus, individuals enter the compartment through being cured out of the active new (A_d,h,a,t_), active, previously treated (R_d,h,a,t_) or failure (F_d,h,a,t_) compartments or through reinfection of patients who had achieved cure from a prior infection (C_d_) but do not progress to active disease. Individuals leave the compartment through reinfection with TB or death (from TB or other causes). Cured compartments are subdivided by drug-susceptibility, HIV-status, and age group.

1. **Additional analyses**
2. Economic Evaluation

We performed a cost-effectiveness analysis from the TB program perspective, calculating the incremental cost-effectiveness ratio (ICER) of TB diagnosis and treatment, measured in U.S. dollars (year 2012) per life year gained (YLG). The cost of diagnostic testing in India was taken from an empiric study reported in the literature [[18](#_ENREF_18)]. Treatment cost was abstracted from the WHO financing report for India in 2012 (using US Dollars) [[97](#_ENREF_97)]. Inflation to 2012 was performed using the World Bank GDP deflator for US Dollars [[98](#_ENREF_98)], and future costs and YLGs were discounted at 3% annually. We assumed that all the cost of all novel tests was similar to that of Xpert. In addition, we considered POC non-sputum NAAT at a price point of $8 per test.

The projected incremental cost per YLG, relative to the existing standard of care, was similar for Xpert and all optimized NAAT tests, ranging from $1400 to $2100 (Table 3). POC non-sputum NAAT – which had the greatest impact on overall TB mortality despite not being able to diagnose MDR-TB – was the most effective and cost-effective option, even assuming the same cost for this test as for Xpert (Supplementary Table E6). MDR-TB treatment accounted for about 40% of all incremental costs in the Xpert-based scenarios.

The cost estimates for all tests (except for POC non-sputum NAAT) are very similar. The estimate for the cost of Xpert per life-year gained exceeds those projected by other studies, even though we only project cost for TB care (not including cost conferred by HIV-treatment) [[18](#_ENREF_18), [99](#_ENREF_99)]. This is again explained by the lower incremental effectiveness of Xpert in our study as compared to prior evaluations that assumed lower levels of empiric diagnosis [[18](#_ENREF_18), [22](#_ENREF_22), [99](#_ENREF_99)]. The cost per life-year gained in our study meets existing thresholds (e.g., cost per life-year gained less than per-capita GDP) for cost-effective interventions in most Southeast Asian countries [[100](#_ENREF_100)]. But even independent of cost-effectiveness, tests targeting pediatric TB and EPTB would likely have a substantial market potential given their impact on incidence and/or mortality, coupled with the lack of good existing diagnostic options in these individuals. Thus, both cost-effectiveness and market considerations may favor the development of such assays, even though their direct effect on TB incidence will be limited.

1. Additional sensitivity analyses

Additional sensitivity analyses were performed to assess variables that have the most impact on the results across different comparisons: diagnostic rate per year in new cases, sensitivity of standard test as well as incremental sensitivity of novel test for PTB detection, proportion never diagnosed and the proportion of patients who progress to primary disease immediately after infection. The difference in the adult EPTB mortality was proportionally similar across the different parameters in the different scenarios and the size of the difference depended on the incremental effect of the individual scenario over the existing standard with the POC non-sputum NAAT having the most substantial effect (Supplementary Figure E1 for the comparison of the effect of NAAT EPTB with the existing standard on adult EPTB mortality).

1. Three-way sensitivity analysis

The three-way sensitivity analysis compared the impact of the existing standard sensitivity for PTB, the incremental sensitivity of a novel test and the diagnostic rate for new cases on mortality from adult extrapulmonary tuberculosis.

The Supplementary Table E7 demonstrates that the diagnostic rate exerts that largest impact on adult EPTB mortality. The impact of the sensitivity of the existing standard and the incremental sensitivity of the novel test are largely dependent on the diagnostic rate. The substantial impact of the diagnostic rate also explains the sizeable improvement in mortality outcomes of the POC non-sputum NAAT as this is the only testing strategy that affects the diagnostic rate in addition to having improved deployability (similar to POC sputum NAAT).

**Supplementary Table E6: Incremental cost per life-year gained**

Incremental cost per life-year saved comparing the different test scenarios over 10 years.

| **Test scenario** | **Total number of new tests*** | **Total number treated** | **Difference in number treated** | **Diagnostic cost (US$)** | **MDR treatment cost (US$)** | **Total treatment cost (US$)^#^** | **Total cost (US$)** | **Incremen-**  **tal life-years gained** | **Incremental cost per life-year gained (ICER)^+^** |
| --- | --- | --- | --- | --- | --- | --- | --- | --- | --- |
| Existing standard | 0 | 1,486 | Reference | 20,507 | 54,196 | 153,758 | 174,265 | Reference | Reference |
| Xpert | 2,276 | 1,448 | -38 | 56,700 | 61,495 | 158,536 | 215,237 | 20 | 2,078 |
| NAAT-Peds | 2,748 | 1,452 | -34 | 64,200 | 64,394 | 161,663 | 225,863 | 27 | 1,934 |
| NAAT-EPTB | 3,538 | 1,457 | -29 | 76,769 | 68,559 | 166,211 | 242,979 | 35 | 1,968 |
| POC sputum NAAT | 7,200 | 1,384 | -102 | 135,039 | 70,004 | 162,728 | 297,767 | 64 | 1,937 |
| POC non-sputum NAAT |  |  |  |  |  |  |  |  |  |
| Cost $8 | 13,941 | 1,508 | 22 | 108,134 | 32,861 | 133,875 | 242,009 | 146 | 465 |
| Cost $19.58 |  |  |  | 248,010 |  |  | 381,885 |  | 1,425 |

^*^Other than smear and other existing tests (e.g., X-ray) assuming that 1 in 10 patients tested has tuberculosis; ^#^Treatment cost first-line therapy: US$67, MDR therapy: US$2,500; ^+^All values are relative to the reference of the existing standard; Abbreviations: POC=point of care; TB= tuberculosis; NAAT=nucleic-acid amplification test; EPTB=extrapulmonary TB

**Supplementary Table E7:**

Three-way sensitivity analysis of the impact of the sensitivity of the existing standard for pulmonary TB (PTB), the incremental sensitivity of a novel test and the diagnostic rate for new cases on adult EPTB mortality in the NAAT EPTB scenario. The table demonstrates that the diagnostic rate exerts that largest impact on adult EPTB mortality.

|  | **Sensitivity novel test for PTB** | | | | |  |
| --- | --- | --- | --- | --- | --- | --- |
| **Sensitivity existing standard for PTB** |  | **0.6** | **0.8** | **0.95** |  | **Diagnostic-rate per year in new cases** |
|  | **0.6** | 30.2 | 23.8 | 20.3 | **1** |  |
|  | **0.8** | - | 19.8 | 17.3 | **1** |  |
|  | **0.6** | 9.7 | 8.1 | 7.3 | **2** |  |
|  | **0.8** | - | 7.2 | 6.7 | **2** |  |
|  | **0.6** | 5.9 | 5.2 | 4.9 | **3** |  |
|  | **0.8** | - | 4.8 | 4.6 | **3** |  |

**Figure Legends:**

**Supplementary Figure E1:**

Absolute difference in extrapulmonary tuberculosis (EPTB) mortality in adults per 100,000 by year 10 if NAAT-EPTB is compared to the existing standard (ES) varying one parameter at the time (base-case: reduction of 0.6 in adult EPTB mortality comparing NAAT-EPTB with the existing standard when all parameters are kept stable). The analysis shows that effect of NAAT EPTB is primarily dependent on reducing transmission of pulmonary TB (PTB) and the sensitivity of the test for existing standard for PTB in conjunction with the rate at which the test is used.

**References**

1. Andrews JR, Noubary F, Walensky RP, Cerda R, Losina E, Horsburgh CR: **Risk of progression to active tuberculosis following reinfection with Mycobacterium tuberculosis**. *Clin Infect Dis* 2012, **54**(6):784-791.

2. Vynnycky E, Fine PE: **The natural history of tuberculosis: the implications of age-dependent risks of disease and the role of reinfection**. *Epidemiol Infect* 1997, **119**(2):183-201.

3. Sutherland I, Svandova E, Radhakrishna S: **The development of clinical tuberculosis following infection with tubercle bacilli. 1. A theoretical model for the development of clinical tuberculosis following infection, linking from data on the risk of tuberculous infection and the incidence of clinical tuberculosis in the Netherlands**. *Tubercle* 1982, **63**(4):255-268.

4. van Leth F, van der Werf MJ, Borgdorff MW: **Prevalence of tuberculous infection and incidence of tuberculosis: a re-assessment of the Styblo rule**. *Bull World Health Organ* 2008, **86**(1):20-26.

5. Joint United Nations Programme on HIV/AIDS (UNAIDS): **Global report: UNAIDS report on the global AIDS epidemic 2012**. In*.* Geneva; 2012.

6. Corbett EL, Charalambous S, Moloi VM, Fielding K, Grant AD, Dye C, De Cock KM, Hayes RJ, Williams BG, Churchyard GJ: **Human immunodeficiency virus and the prevalence of undiagnosed tuberculosis in African gold miners**. *Am J Respir Crit Care Med* 2004, **170**(6):673-679.

7. Antonucci G, Girardi E, Armignacco O, Salmaso S, Ippolito G: **Tuberculosis in HIV-infected subjects in Italy: a multicentre study. The Gruppo Italiano di Studio Tubercolosi e AIDS**. *AIDS* 1992, **6**(9):1007-1013.

8. Gilks CF, Godfrey-Faussett P, Batchelor BI, Ojoo JC, Ojoo SJ, Brindle RJ, Paul J, Kimari J, Bruce MC, Bwayo J *et al*: **Recent transmission of tuberculosis in a cohort of HIV-1-infected female sex workers in Nairobi, Kenya**. *AIDS* 1997, **11**(7):911-918.

9. Holmes CB, Wood R, Badri M, Zilber S, Wang B, Maartens G, Zheng H, Lu Z, Freedberg KA, Losina E: **CD4 decline and incidence of opportunistic infections in Cape Town, South Africa: implications for prophylaxis and treatment**. *J Acquir Immune Defic Syndr* 2006, **42**(4):464-469.

10. Harries AD, Hargreaves NJ, Graham SM, Mwansambo C, Kazembe P, Broadhead RL, Maher D, Salaniponi FM: **Childhood tuberculosis in Malawi: nationwide case-finding and treatment outcomes**. *Int J Tuberc Lung Dis* 2002, **6**(5):424-431.

11. Hesseling AC, Cotton MF, Jennings T, Whitelaw A, Johnson LF, Eley B, Roux P, Godfrey-Faussett P, Schaaf HS: **High incidence of tuberculosis among HIV-infected infants: evidence from a South African population-based study highlights the need for improved tuberculosis control strategies**. *Clin Infect Dis* 2009, **48**(1):108-114.

12. Marais BJ, Gie RP, Schaaf HS, Hesseling AC, Obihara CC, Starke JJ, Enarson DA, Donald PR, Beyers N: **The natural history of childhood intra-thoracic tuberculosis: a critical review of literature from the pre-chemotherapy era**. *Int J Tuberc Lung Dis* 2004, **8**(4):392-402.

13. Zar HJ, Hanslo D, Apolles P, Swingler G, Hussey G: **Induced sputum versus gastric lavage for microbiological confirmation of pulmonary tuberculosis in infants and young children: a prospective study**. *Lancet* 2005, **365**(9454):130-134.

14. Chaisson RE, Schecter GF, Theuer CP, Rutherford GW, Echenberg DF, Hopewell PC: **Tuberculosis in patients with the acquired immunodeficiency syndrome. Clinical features, response to therapy, and survival**. *Am Rev Respir Dis* 1987, **136**(3):570-574.

15. Jones BE, Young SM, Antoniskis D, Davidson PT, Kramer F, Barnes PF: **Relationship of the manifestations of tuberculosis to CD4 cell counts in patients with human immunodeficiency virus infection**. *Am Rev Respir Dis* 1993, **148**(5):1292-1297.

16. Peter JG, Theron G, Singh N, Singh A, Dheda K: **Sputum induction to aid diagnosis of smear-negative or sputum-scarce tuberculosis in adults in HIV-endemic settings**. *Eur Respir J* 2014, **43**(1):185-194.

17. Keeler E, Perkins MD, Small P, Hanson C, Reed S, Cunningham J, Aledort JE, Hillborne L, Rafael ME, Girosi F *et al*: **Reducing the global burden of tuberculosis: the contribution of improved diagnostics**. *Nature* 2006, **444 Suppl 1**:49-57.

18. Vassall A, van Kampen S, Sohn H, Michael JS, John KR, den Boon S, Davis JL, Whitelaw A, Nicol MP, Gler MT *et al*: **Rapid diagnosis of tuberculosis with the Xpert MTB/RIF assay in high burden countries: a cost-effectiveness analysis**. *PLoS Med* 2011, **8**(11):e1001120.

19. Kapoor SK, Raman AV, Sachdeva KS, Satyanarayana S: **How did the TB patients reach DOTS services in Delhi? A study of patient treatment seeking behavior**. *PLoS One* 2012, **7**(8):e42458.

20. Storla DG, Yimer S, Bjune GA: **A systematic review of delay in the diagnosis and treatment of tuberculosis**. *BMC Public Health* 2008, **8**:15.

21. Corbett EL, Watt CJ, Walker N, Maher D, Williams BG, Raviglione MC, Dye C: **The growing burden of tuberculosis: global trends and interactions with the HIV epidemic**. *Arch Intern Med* 2003, **163**(9):1009-1021.

22. Boehme CC, Nicol MP, Nabeta P, Michael JS, Gotuzzo E, Tahirli R, Gler MT, Blakemore R, Worodria W, Gray C *et al*: **Feasibility, diagnostic accuracy, and effectiveness of decentralised use of the Xpert MTB/RIF test for diagnosis of tuberculosis and multidrug resistance: a multicentre implementation study**. *Lancet* 2011, **377**(9776):1495-1505.

23. Leutscher P, Madsen G, Erlandsen M, Veirum J, Ladefoged K, Thomsen V, Wejse C, Hilberg O: **Demographic and clinical characteristics in relation to patient and health system delays in a tuberculosis low-incidence country**. *Scand J Infect Dis* 2012, **44**(1):29-36.

24. Claassens MM, du Toit E, Dunbar R, Lombard C, Enarson DA, Beyers N, Borgdorff MW: **Tuberculosis patients in primary care do not start treatment. What role do health system delays play?** *Int J Tuberc Lung Dis* 2013, **17**(5):603-607.

25. World Health Organization: **Global tuberculosis report 2012**. In*.* Geneva 2012: 1-282.

26. Behr MA, Warren SA, Salamon H, Hopewell PC, Ponce de Leon A, Daley CL, Small PM: **Transmission of Mycobacterium tuberculosis from patients smear-negative for acid-fast bacilli**. *Lancet* 1999, **353**(9151):444-449.

27. Fennelly KP, Jones-Lopez EC, Ayakaka I, Kim S, Menyha H, Kirenga B, Muchwa C, Joloba M, Dryden-Peterson S, Reilly N *et al*: **Variability of infectious aerosols produced during coughing by patients with pulmonary tuberculosis**. *Am J Respir Crit Care Med* 2012, **186**(5):450-457.

28. **HIV/AIDS in India** [http://www.worldbank.org/en/news/feature/2012/07/10/hiv-aids-india]

29. Pym AS, Saint-Joanis B, Cole ST: **Effect of katG mutations on the virulence of Mycobacterium tuberculosis and the implication for transmission in humans**. *Infect Immun* 2002, **70**(9):4955-4960.

30. van Soolingen D, de Haas PE, van Doorn HR, Kuijper E, Rinder H, Borgdorff MW: **Mutations at amino acid position 315 of the katG gene are associated with high-level resistance to isoniazid, other drug resistance, and successful transmission of Mycobacterium tuberculosis in the Netherlands**. *J Infect Dis* 2000, **182**(6):1788-1790.

31. Cohen T, Murray M: **Modeling epidemics of multidrug-resistant M. tuberculosis of heterogeneous fitness**. *Nat Med* 2004, **10**(10):1117-1121.

32. Billington OJ, McHugh TD, Gillespie SH: **Physiological cost of rifampin resistance induced in vitro in Mycobacterium tuberculosis**. *Antimicrobial agents and chemotherapy* 1999, **43**(8):1866-1869.

33. Gagneux S, Long CD, Small PM, Van T, Schoolnik GK, Bohannan BJ: **The competitive cost of antibiotic resistance in Mycobacterium tuberculosis**. *Science* 2006, **312**(5782):1944-1946.

34. Hanrahan CF, Theron G, Bassett J, Dheda K, Scott L, Stevens W, Sanne I, Van Rie A: **Xpert MTB/RIF as a measure of sputum bacillary burden. Variation by HIV status and immunosuppression**. *Am J Respir Crit Care Med* 2014, **189**(11):1426-1434.

35. Tostmann A, Kik SV, Kalisvaart NA, Sebek MM, Verver S, Boeree MJ, van Soolingen D: **Tuberculosis transmission by patients with smear-negative pulmonary tuberculosis in a large cohort in the Netherlands**. *Clin Infect Dis* 2008, **47**(9):1135-1142.

36. Espinal MA, Perez EN, Baez J, Henriquez L, Fernandez K, Lopez M, Olivo P, Reingold AL: **Infectiousness of Mycobacterium tuberculosis in HIV-1-infected patients with tuberculosis: a prospective study**. *Lancet* 2000, **355**(9200):275-280.

37. Marais BJ, Gie RP, Schaaf HS, Hesseling AC, Obihara CC, Nelson LJ, Enarson DA, Donald PR, Beyers N: **The clinical epidemiology of childhood pulmonary tuberculosis: a critical review of literature from the pre-chemotherapy era**. *Int J Tuberc Lung Dis* 2004, **8**(3):278-285.

38. Peto HM, Pratt RH, Harrington TA, LoBue PA, Armstrong LR: **Epidemiology of extrapulmonary tuberculosis in the United States, 1993-2006**. *Clin Infect Dis* 2009, **49**(9):1350-1357.

39. Marais BJ, Gie RP, Schaaf HS, Beyers N, Donald PR, Starke JR: **Childhood pulmonary tuberculosis: old wisdom and new challenges**. *Am J Respir Crit Care Med* 2006, **173**(10):1078-1090.

40. Ferebee SH: **Controlled chemoprophylaxis trials in tuberculosis. A general review**. *Bibliotheca tuberculosea* 1970, **26**:28-106.

41. Grzybowski S, Enarson D: **[Results in pulmonary tuberculosis patients under various treatment program conditions]**. *Bulletin of the International Union against Tuberculosis* 1978, **53**(2):70-75.

42. Dye C, Garnett GP, Sleeman K, Williams BG: **Prospects for worldwide tuberculosis control under the WHO DOTS strategy. Directly observed short-course therapy**. *Lancet* 1998, **352**(9144):1886-1891.

43. Wong EB, Omar T, Setlhako GJ, Osih R, Feldman C, Murdoch DM, Martinson NA, Bangsberg DR, Venter WD: **Causes of death on antiretroviral therapy: a post-mortem study from South Africa**. *PLoS One* 2012, **7**(10):e47542.

44. Etard JF, Ndiaye I, Thierry-Mieg M, Gueye NF, Gueye PM, Laniece I, Dieng AB, Diouf A, Laurent C, Mboup S *et al*: **Mortality and causes of death in adults receiving highly active antiretroviral therapy in Senegal: a 7-year cohort study**. *AIDS* 2006, **20**(8):1181-1189.

45. Davis JL, Cattamanchi A, Cuevas LE, Hopewell PC, Steingart KR: **Diagnostic accuracy of same-day microscopy versus standard microscopy for pulmonary tuberculosis: a systematic review and meta-analysis**. *Lancet Infect Dis* 2013, **13**(2):147-154.

46. Mase SR, Ramsay A, Ng V, Henry M, Hopewell PC, Cunningham J, Urbanczik R, Perkins MD, Aziz MA, Pai M: **Yield of serial sputum specimen examinations in the diagnosis of pulmonary tuberculosis: a systematic review**. *Int J Tuberc Lung Dis* 2007, **11**(5):485-495.

47. Geng E, Kreiswirth B, Burzynski J, Schluger NW: **Clinical and radiographic correlates of primary and reactivation tuberculosis: a molecular epidemiology study**. *JAMA* 2005, **293**(22):2740-2745.

48. Dinnes J, Deeks J, Kunst H, Gibson A, Cummins E, Waugh N, Drobniewski F, Lalvani A: **A systematic review of rapid diagnostic tests for the detection of tuberculosis infection**. *Health Technol Assess* 2007, **11**(3):1-314.

49. Kennedy DH, Fallon RJ: **Tuberculous meningitis**. *JAMA* 1979, **241**(3):264-268.

50. Lau SK, Wei WI, Hsu C, Engzell UC: **Efficacy of fine needle aspiration cytology in the diagnosis of tuberculous cervical lymphadenopathy**. *The Journal of laryngology and otology* 1990, **104**(1):24-27.

51. Marais S, Thwaites G, Schoeman JF, Torok ME, Misra UK, Prasad K, Donald PR, Wilkinson RJ, Marais BJ: **Tuberculous meningitis: a uniform case definition for use in clinical research**. *Lancet Infect Dis* 2010, **10**(11):803-812.

52. Steingart KR, Sohn H, Schiller I, Kloda LA, Boehme CC, Pai M, Dendukuri N: **Xpert(R) MTB/RIF assay for pulmonary tuberculosis and rifampicin resistance in adults**. *Cochrane Database Syst Rev* 2013, **1**:CD009593.

53. Friedrich SO, von Groote-Bidlingmaier F, Diacon AH: **Xpert MTB/RIF assay for diagnosis of pleural tuberculosis**. *J Clin Microbiol* 2011, **49**(12):4341-4342.

54. Hillemann D, Ruesch-Gerdes S, Boehme C, Richter E: **Rapid Molecular Detection of Extrapulmonary Tuberculosis by the Automated GeneXpert MTB/RIF System**. *J Clin Microbiol* 2011, **49**(4):1202-1205.

55. Moure R, Martin R, Alcaide F: **Effectiveness of an integrated real-time PCR method for detection of the Mycobacterium tuberculosis complex in smear-negative extrapulmonary samples in an area of low tuberculosis prevalence**. *J Clin Microbiol* 2012, **50**(2):513-515.

56. Tortoli E, Russo C, Piersimoni C, Mazzola E, Dal Monte P, Pascarella M, Borroni E, Mondo A, Piana F, Scarparo C *et al*: **Clinical validation of Xpert MTB/RIF for the diagnosis of extrapulmonary tuberculosis**. *Eur Respir J* 2012, **40**(2):442-447.

57. Chang K, Lu W, Wang J, Zhang K, Jia S, Li F, Deng S, Chen M: **Rapid and effective diagnosis of tuberculosis and rifampicin resistance with Xpert MTB/RIF assay: A meta-analysis**. *J Infect* 2012, **64**(6):580-588.

58. Ling DI, Zwerling AA, Pai M: **GenoType MTBDR assays for the diagnosis of multidrug-resistant tuberculosis: a meta-analysis**. *Eur Respir J* 2008, **32**(5):1165-1174.

59. Feng Y, Liu S, Wang Q, Wang L, Tang S, Wang J, Lu W: **Rapid Diagnosis of Drug Resistance to Fluoroquinolones, Amikacin, Capreomycin, Kanamycin and Ethambutol Using Genotype MTBDRsl Assay: A Meta-Analysis**. *PLoS One* 2013, **8**(2):e55292.

60. Hillemann D, Rusch-Gerdes S, Richter E: **Feasibility of the GenoType MTBDRsl assay for fluoroquinolone, amikacin-capreomycin, and ethambutol resistance testing of Mycobacterium tuberculosis strains and clinical specimens**. *J Clin Microbiol* 2009, **47**(6):1767-1772.

61. Said HM, Kock MM, Ismail NA, Baba K, Omar SV, Osman AG, Hoosen AA, Ehlers MM: **Evaluation of the GenoType(R) MTBDRsl assay for susceptibility testing of second-line anti-tuberculosis drugs**. *Int J Tuberc Lung Dis* 2012, **16**(1):104-109.

62. Botha E, Den Boon S, Verver S, Dunbar R, Lawrence KA, Bosman M, Enarson DA, Toms I, Beyers N: **Initial default from tuberculosis treatment: how often does it happen and what are the reasons?** *Int J Tuberc Lung Dis* 2008, **12**(7):820-823.

63. Khan MS, Khan S, Godfrey-Faussett P: **Default during TB diagnosis: quantifying the problem**. *Trop Med Int Health* 2009, **14**(12):1437-1441.

64. [No author listed]: **A controlled trial of 6 months' chemotherapy in pulmonary tuberculosis. Final report: results during the 36 months after the end of chemotherapy and beyond. British Thoracic Society**. *British journal of diseases of the chest* 1984, **78**(4):330-336.

65. Lew W, Pai M, Oxlade O, Martin D, Menzies D: **Initial drug resistance and tuberculosis treatment outcomes: systematic review and meta-analysis**. *Ann Intern Med* 2008, **149**(2):123-134.

66. Menzies D, Benedetti A, Paydar A, Martin I, Royce S, Pai M, Vernon A, Lienhardt C, Burman W: **Effect of duration and intermittency of rifampin on tuberculosis treatment outcomes: a systematic review and meta-analysis**. *PLoS Med* 2009, **6**(9):e1000146.

67. Espinal MA, Kim SJ, Suarez PG, Kam KM, Khomenko AG, Migliori GB, Baez J, Kochi A, Dye C, Raviglione MC: **Standard short-course chemotherapy for drug-resistant tuberculosis: treatment outcomes in 6 countries**. *JAMA* 2000, **283**(19):2537-2545.

68. Seung KJ, Gelmanova IE, Peremitin GG, Golubchikova VT, Pavlova VE, Sirotkina OB, Yanova GV, Strelis AK: **The effect of initial drug resistance on treatment response and acquired drug resistance during standardized short-course chemotherapy for tuberculosis**. *Clin Infect Dis* 2004, **39**(9):1321-1328.

69. **Primary multidrug-resistant tuberculosis--Ivanovo Oblast, Russia, 1999**. *MMWR Morb Mortal Wkly Rep* 1999, **48**(30):661-664.

70. Menzies D, Benedetti A, Paydar A, Royce S, Madhukar P, Burman W, Vernon A, Lienhardt C: **Standardized treatment of active tuberculosis in patients with previous treatment and/or with mono-resistance to isoniazid: a systematic review and meta-analysis**. *PLoS Med* 2009, **6**(9):e1000150.

71. Hong Kong Chest-British Medical Research Council: **Controlled trial of four twice-weekly regimens and a daily regimen all given for 6 months for pulmonary tuberculosis**. *Lancet* 1981(317):171–174.

72. Migliori GB, Espinal M, Danilova ID, Punga VV, Grzemska M, Raviglione MC: **Frequency of recurrence among MDR-tB cases 'successfully' treated with standardised short-course chemotherapy**. *Int J Tuberc Lung Dis* 2002, **6**(10):858-864.

73. Jones-Lopez EC, Ayakaka I, Levin J, Reilly N, Mumbowa F, Dryden-Peterson S, Nyakoojo G, Fennelly K, Temple B, Nakubulwa S *et al*: **Effectiveness of the standard WHO recommended retreatment regimen (category II) for tuberculosis in Kampala, Uganda: a prospective cohort study**. *PLoS Med* 2011, **8**(3):e1000427.

74. Jacobson KR, Theron D, Victor TC, Streicher EM, Warren RM, Murray MB: **Treatment outcomes of isoniazid-resistant tuberculosis patients, Western Cape Province, South Africa**. *Clin Infect Dis* 2011, **53**(4):369-372.

75. Bang D, Andersen PH, Andersen AB, Thomsen VO: **Isoniazid-resistant tuberculosis in Denmark: mutations, transmission and treatment outcome**. *J Infect* 2010, **60**(6):452-457.

76. Cattamanchi A, Dantes RB, Metcalfe JZ, Jarlsberg LG, Grinsdale J, Kawamura LM, Osmond D, Hopewell PC, Nahid P: **Clinical characteristics and treatment outcomes of patients with isoniazid-monoresistant tuberculosis**. *Clin Infect Dis* 2009, **48**(2):179-185.

77. Sonnenberg P, Murray J, Shearer S, Glynn JR, Kambashi B, Godfrey-Faussett P: **Tuberculosis treatment failure and drug resistance--same strain or reinfection?** *Trans R Soc Trop Med Hyg* 2000, **94**(6):603-607.

78. Tahaoglu K, Torun T, Sevim T, Atac G, Kir A, Karasulu L, Ozmen I, Kapakli N: **The treatment of multidrug-resistant tuberculosis in Turkey**. *N Engl J Med* 2001, **345**(3):170-174.

79. Yew WW, Chan CK, Chau CH, Tam CM, Leung CC, Wong PC, Lee J: **Outcomes of patients with multidrug-resistant pulmonary tuberculosis treated with ofloxacin/levofloxacin-containing regimens**. *Chest* 2000, **117**(3):744-751.

80. Mitnick C, Bayona J, Palacios E, Shin S, Furin J, Alcantara F, Sanchez E, Sarria M, Becerra M, Fawzi MC *et al*: **Community-based therapy for multidrug-resistant tuberculosis in Lima, Peru**. *N Engl J Med* 2003, **348**(2):119-128.

81. Orenstein EW, Basu S, Shah NS, Andrews JR, Friedland GH, Moll AP, Gandhi NR, Galvani AP: **Treatment outcomes among patients with multidrug-resistant tuberculosis: systematic review and meta-analysis**. *Lancet Infect Dis* 2009, **9**(3):153-161.

82. Cavanaugh JS, Kazennyy BY, Nguyen ML, Kiryanova EV, Vitek E, Khorosheva TM, Nemtsova E, Cegielski JP: **Outcomes and follow-up of patients treated for multidrug-resistant tuberculosis in Orel, Russia, 2002-2005**. *Int J Tuberc Lung Dis* 2012, **16**(8):1069-1074.

83. Lee J, Lim HJ, Cho YJ, Park YS, Lee SM, Yang SC, Yoo CG, Kim YW, Han SK, Yim JJ: **Recurrence after successful treatment among patients with multidrug-resistant tuberculosis**. *Int J Tuberc Lung Dis* 2011, **15**(10):1331-1333.

84. Franke MF, Appleton SC, Mitnick CD, Furin JJ, Bayona J, Chalco K, Shin S, Murray M, Becerra MC: **Aggressive Regimens for Multidrug-Resistant Tuberculosis Reduce Recurrence**. *Clin Infect Dis* 2013.

85. Ershova JV, Kurbatova EV, Moonan PK, Cegielski JP: **Acquired resistance to second-line drugs among persons with tuberculosis in the United States**. *Clin Infect Dis* 2012, **55**(12):1600-1607.

86. Jacobson KR, Tierney DB, Jeon CY, Mitnick CD, Murray MB: **Treatment outcomes among patients with extensively drug-resistant tuberculosis: systematic review and meta-analysis**. *Clin Infect Dis* 2010, **51**(1):6-14.

87. Jeon CY, Hwang SH, Min JH, Prevots DR, Goldfeder LC, Lee H, Eum SY, Jeon DS, Kang HS, Kim JH *et al*: **Extensively drug-resistant tuberculosis in South Korea: risk factors and treatment outcomes among patients at a tertiary referral hospital**. *Clin Infect Dis* 2008, **46**(1):42-49.

88. Kwon YS, Kim YH, Suh GY, Chung MP, Kim H, Kwon OJ, Choi YS, Kim K, Kim J, Shim YM *et al*: **Treatment outcomes for HIV-uninfected patients with multidrug-resistant and extensively drug-resistant tuberculosis**. *Clin Infect Dis* 2008, **47**(4):496-502.

89. Falzon D, Gandhi N, Migliori GB, Sotgiu G, Cox H, Holtz TH, Hollm-Delgado MG, Keshavjee S, Deriemer K, Centis R *et al*: **Resistance to fluoroquinolones and second-line injectable drugs: impact on MDR-TB outcomes**. *Eur Respir J* 2012.

90. Heym B, Stavropoulos E, Honore N, Domenech P, Saint-Joanis B, Wilson TM, Collins DM, Colston MJ, Cole ST: **Effects of overexpression of the alkyl hydroperoxide reductase AhpC on the virulence and isoniazid resistance of Mycobacterium tuberculosis**. *Infect Immun* 1997, **65**(4):1395-1401.

91. Jenkins HE, Zignol M, Cohen T: **Quantifying the burden and trends of isoniazid resistant tuberculosis, 1994-2009**. *PLoS One* 2011, **6**(7):e22927.

92. Cohn DL, Bustreo F, Raviglione MC: **Drug-resistant tuberculosis: review of the worldwide situation and the WHO/IUATLD Global Surveillance Project. International Union Against Tuberculosis and Lung Disease**. *Clin Infect Dis* 1997, **24 Suppl 1**:S121-130.

93. Paramasivan CN, Chandrasekaran V, Santha T, Sudarsanam NM, Prabhakar R: **Bacteriological investigations for short-course chemotherapy under the tuberculosis programme in two districts of India**. *Tuber Lung Dis* 1993, **74**(1):23-27.

94. Enarson D, Rouillon A: **The epidemiological basis of tuberculosis control**. London, UK: Chapman and Hall; 1994.

95. **Guidelines for intensified tuberculosis case-finding and isoniazid preventive therapy for people living with HIV in resource-constrained settings** [http://whqlibdoc.who.int/publications/2011/9789241500708_eng.pdf]

96. Boehme CC, Nabeta P, Hillemann D, Nicol MP, Shenai S, Krapp F, Allen J, Tahirli R, Blakemore R, Rustomjee R *et al*: **Rapid molecular detection of tuberculosis and rifampin resistance**. *N Engl J Med* 2010, **363**(11):1005-1015.

97. **Tuberculosis finance profile - India**

98. **Inflation, GDP deflator (annual %)** [http://data.worldbank.org/indicator/NY.GDP.DEFL.KD.ZG]

99. Menzies NA, Cohen T, Lin HH, Murray M, Salomon JA: **Population health impact and cost-effectiveness of tuberculosis diagnosis with Xpert MTB/RIF: a dynamic simulation and economic evaluation**. *PLoS Med* 2012, **9**(11):e1001347.

100. World Health Organization: **CHOosing Interventions that are Cost Effective (WHO-CHOICE)**. In*.*
