# Supplementary figures and images for: Modeling the impact of novel diagnostic tests on pediatric and extrapulmonary tuberculosis
Source: BMC Infect Dis. 2014 Sep 3;14:477. doi: 10.1186/1471-2334-14-477 (PMC4168123; doi:10.1186/1471-2334-14-477)

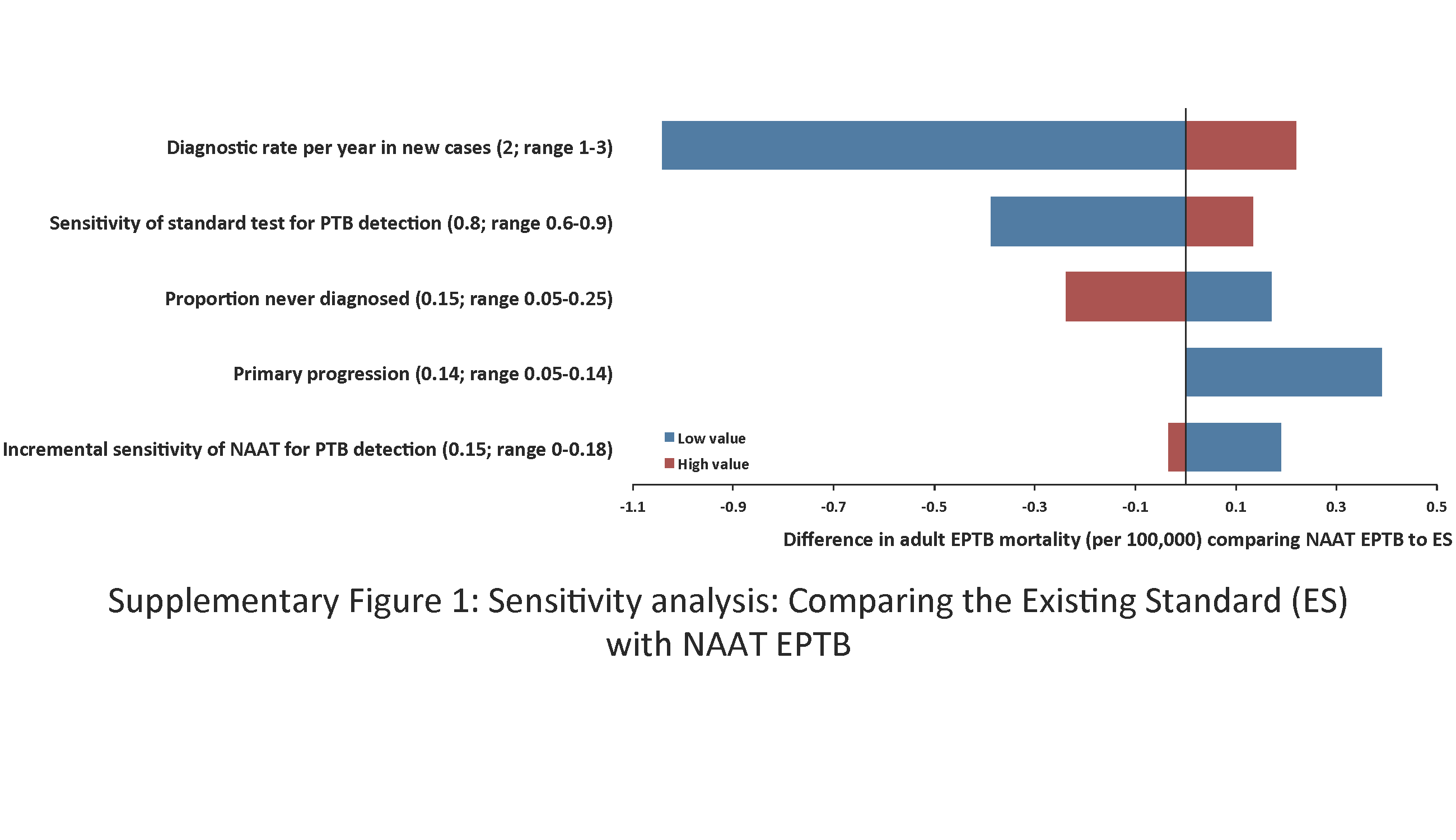

Supplement: Supplementary file 2 — Additional file 2: Figure S1: Sensitivity analysis: comparing the Existing Standard (ES) with NAAT EPTB. (TIFF 315 KB) [file 12879_2014_3797_MOESM2_ESM.tiff]

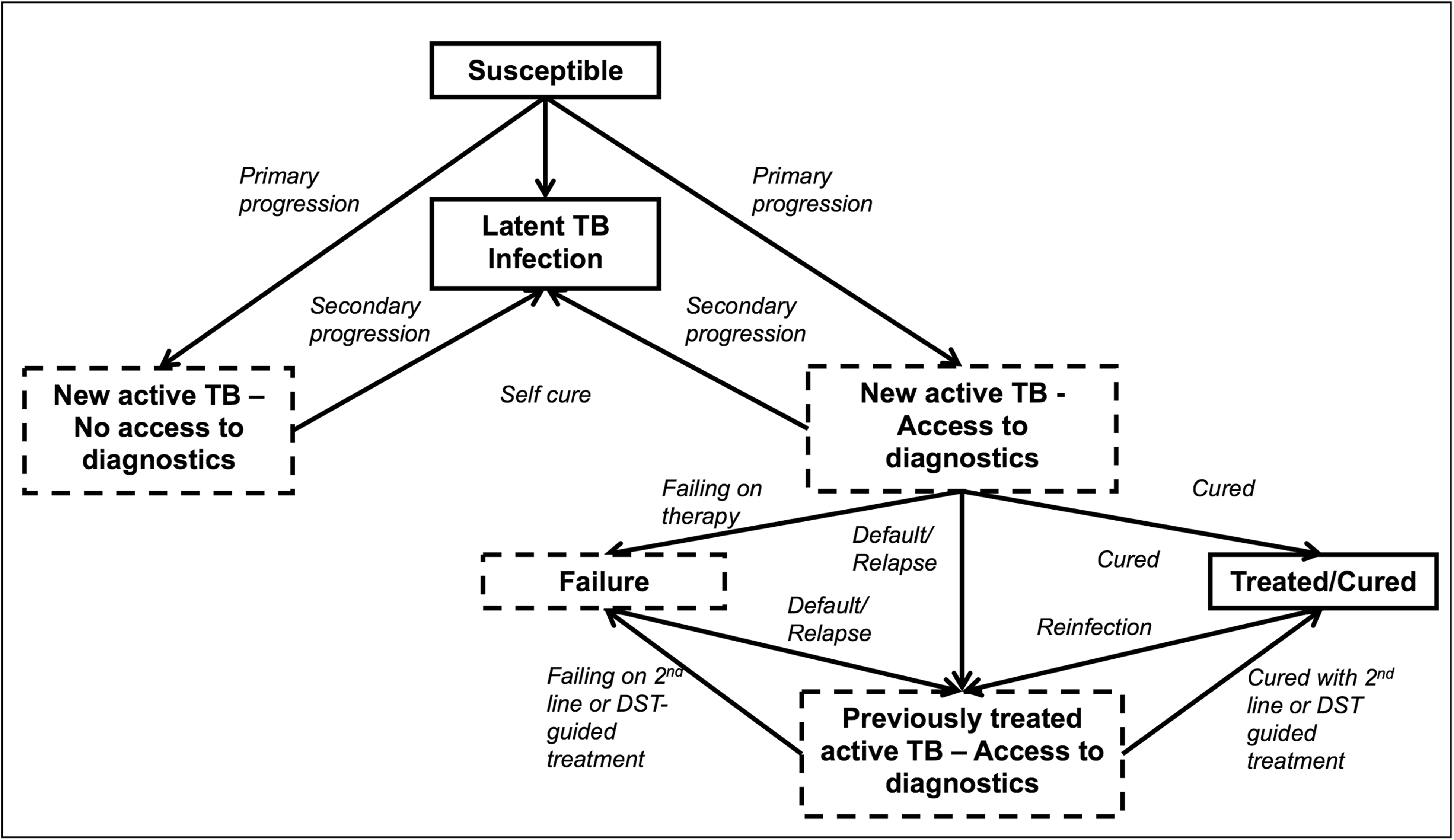

Supplement: Supplementary file 3 — Authors’ original file for figure 1 [file 12879_2014_3797_MOESM3_ESM.tiff]

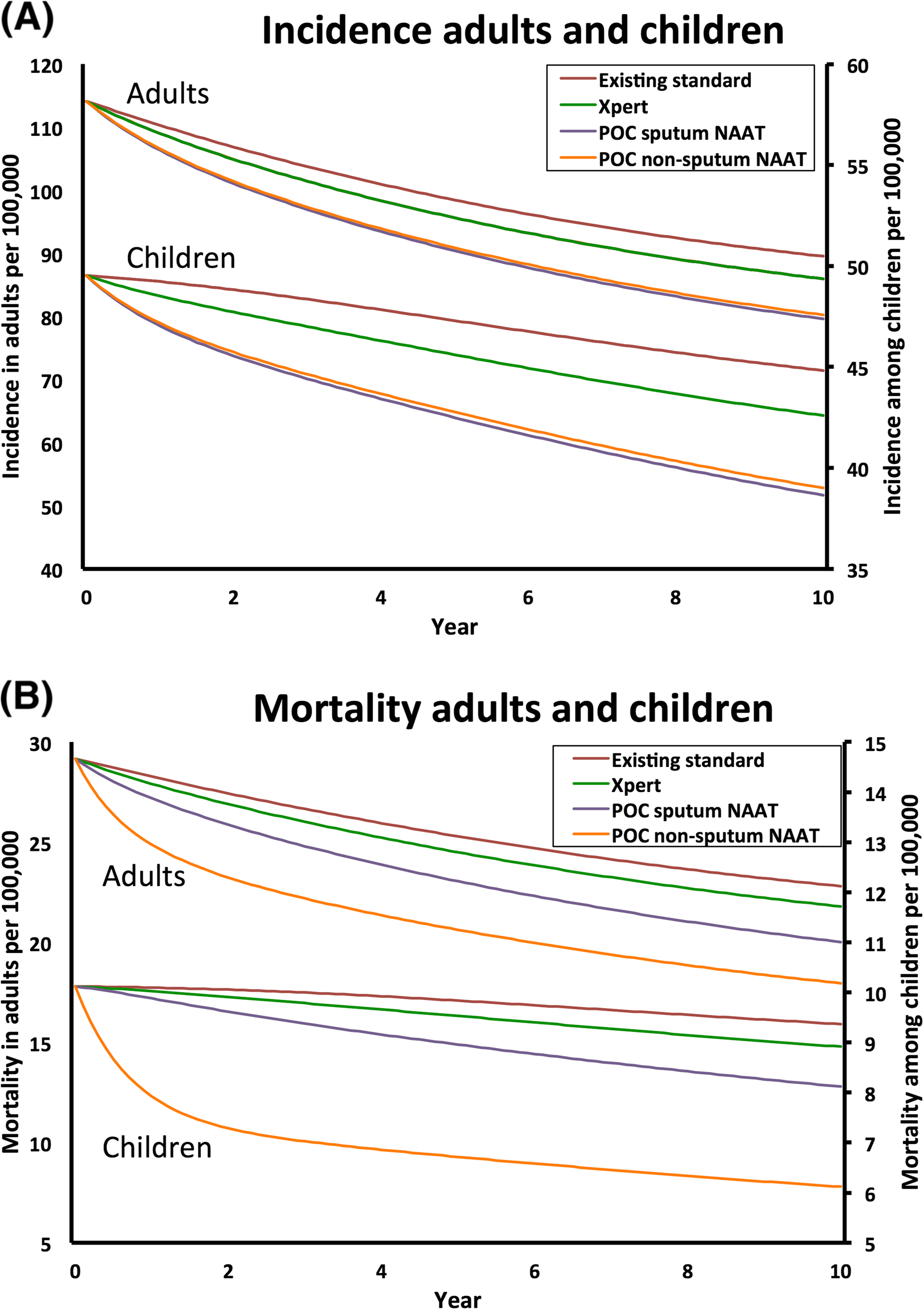

Supplement: Supplementary file 4 — Authors’ original file for figure 2 [file 12879_2014_3797_MOESM4_ESM.tif]

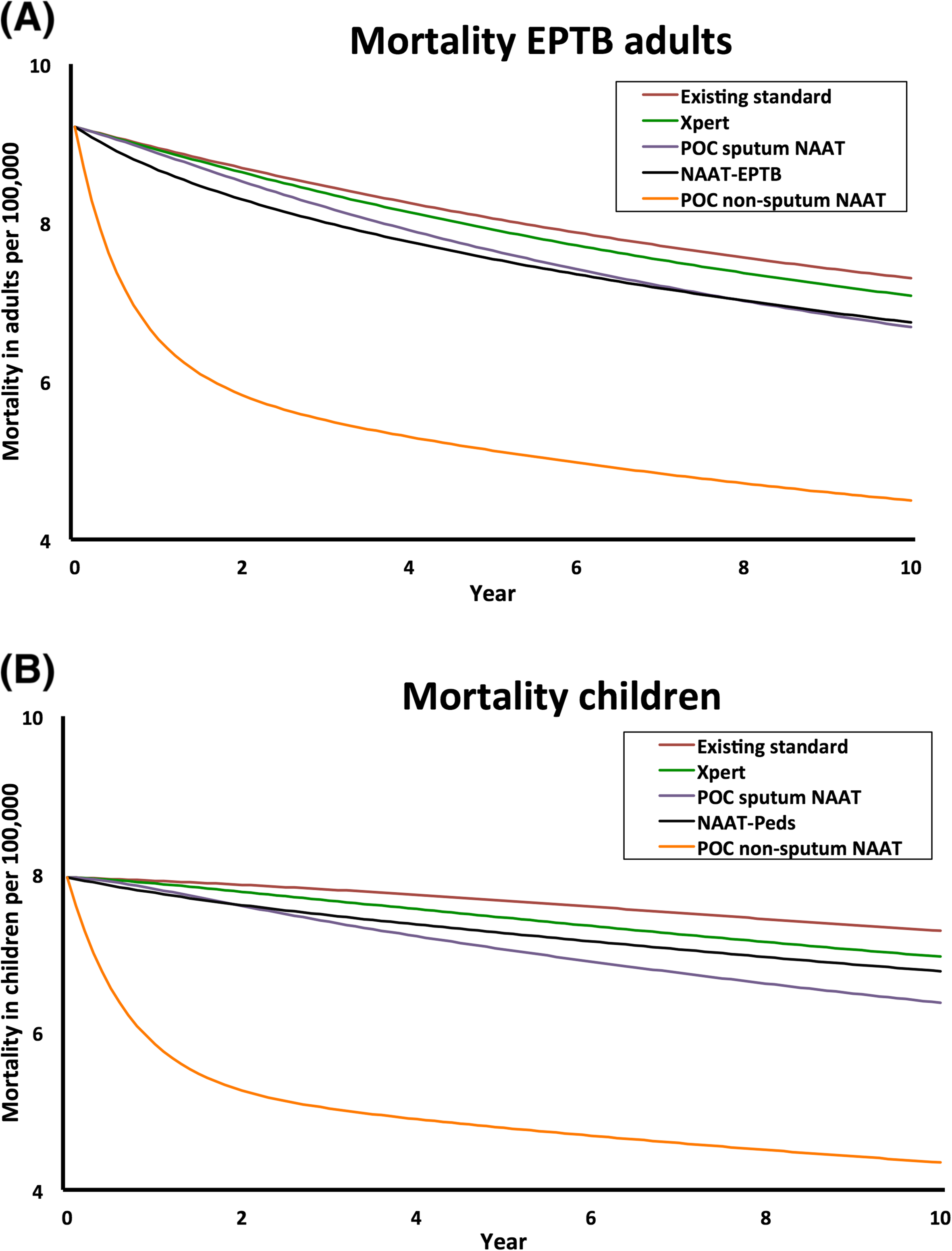

Supplement: Supplementary file 5 — Authors’ original file for figure 3 [file 12879_2014_3797_MOESM5_ESM.tif]

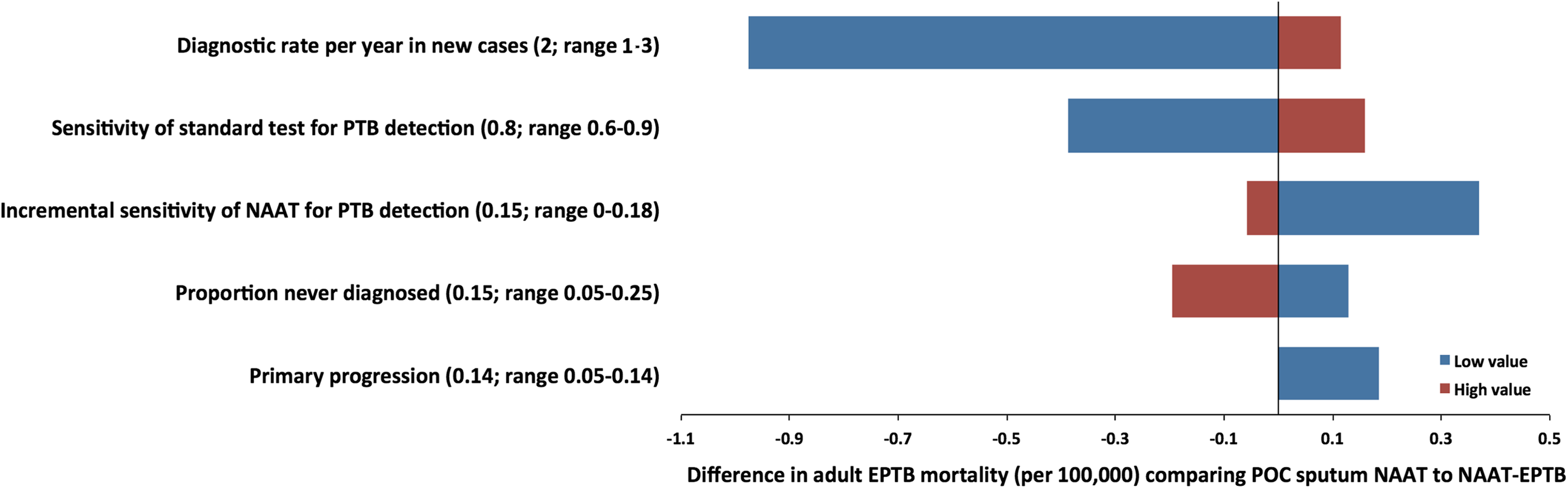

Supplement: Supplementary file 6 — Authors’ original file for figure 4 [file 12879_2014_3797_MOESM6_ESM.tiff]

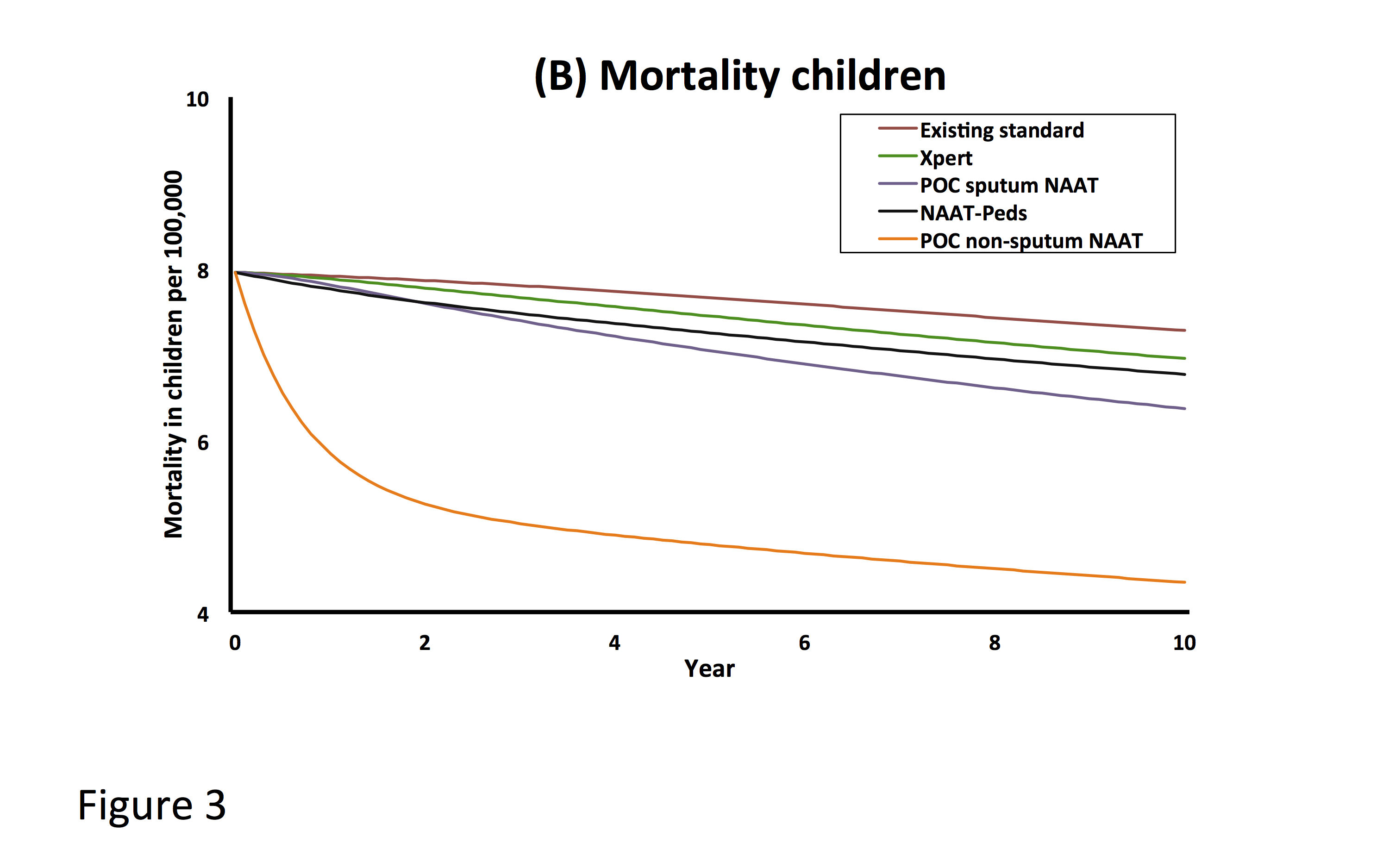

Supplement: Supplementary file 7 — Authors’ original file for figure 5 [file 12879_2014_3797_MOESM7_ESM.tiff]

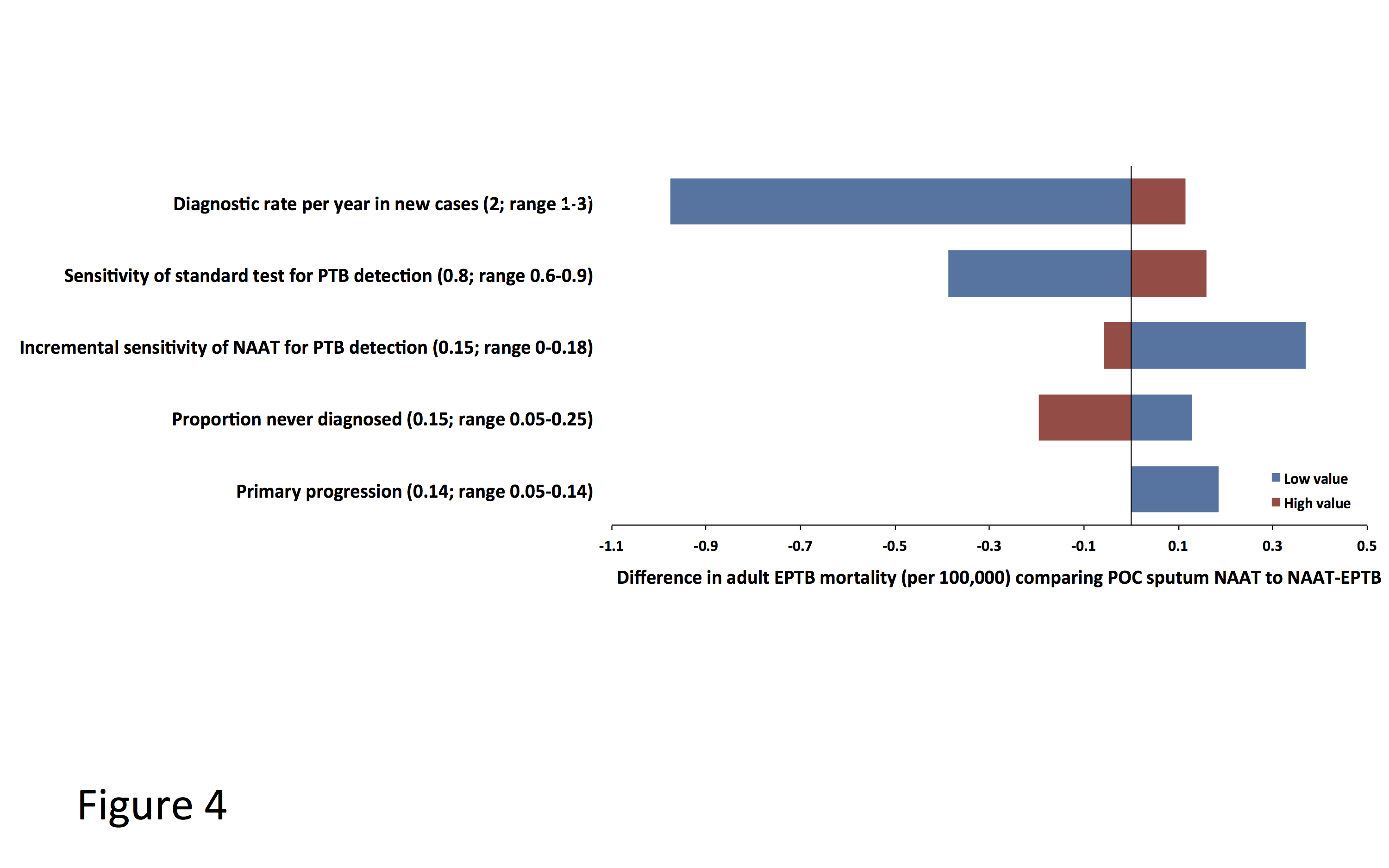

Supplement: Supplementary file 8 — Authors’ original file for figure 6 [file 12879_2014_3797_MOESM8_ESM.tiff]
